# The basic route of nuclear‐targeted transport of IGF‐1/IGF‐1R and potential biological functions in intestinal epithelial cells

**DOI:** 10.1111/cpr.13030

**Published:** 2021-05-01

**Authors:** Ming Xiu, Xia Huan, Yang Ou, Sha Ying, Jianmeng Wang

**Affiliations:** ^1^ Department of Intensive care unit The first hospital of Jilin University Changchun China; ^2^ Department of Respiratory Medicine The First Hospital of Jilin University Changchun China; ^3^ The department of Geriatris The first hospital of Jilin University Changchun China

**Keywords:** cell proliferation, IGF‐1, IGF‐1R, nuclear localization, signalling pathway

## Abstract

**Objectives:**

Insulin‐like growth factor (IGF‐1) plays an important role in many biological processes in the intestinal tract. However, the cellular behaviour and characteristics of IGF‐1/IGF‐1R in intestinal cells remain unclear.

**Materials and Methods:**

A series of techniques (such as indirect immunofluorescence, co‐localization and Western blot) have been used to systematically study the cellular behaviour of IGF‐1/IGF‐1R on intestinal cells.

**Results:**

We found that IGF‐1 can not only internalize into the cytoplasm, but also transport into the cell nuclei. We systematically studied the detailed molecular pathways of IGF‐1/IGF‐1R’s nuclear translocation. We found that IGF‐1R underwent clathrin‐mediated endocytosis into cells and then entered into Rab‐5‐positive endosomes. Dynein/dynactin were used as motors to drive Rab‐5‐positive endosomes carrying IGF‐1R (cargo molecule) to Golgi apparatus (transit station) along the surface of the microtubule. IGF‐1 and/or IGF‐1R entered the cell nuclei through NPC (nuclear pore complex), a process mediated by NUP358. Further study indicated that nuclear localization of IGF‐1 and/or IGF‐1R promoted cell proliferation and increased the nuclear residence time of signalling molecules activated by IGF‐1. Further experiments showed that IGF‐1R may regulate the transcription of genes in the cell nuclei, indicating that nuclear‐localized IGF‐1R plays an important in cell proliferation.

**Conclusions:**

In short, we revealed the molecular mechanism by which IGF‐1/IGF‐1R transports into the cell nuclei of intestinal cells. More importantly, the current work showed that the nuclear‐localized IGF‐1R has important biological functions.

## Graphical abstract

Traditional research believes that IGF‐1 only trigger the intracellular signaling on the cell surface. However, in the current research, in intestinal cells, IGF‐1R can enter into the nucleus from the cell membrane. Therefore, we first revealed the molecular mechanism of IGF‐1R entering into the cell nuclei. On this basis, further studies have revealed that nuclear‐localized IGF‐1R can Regulate cell proliferation.

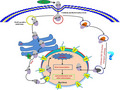

## 1 INTRODUCTION

Insulin‐like growth factor (IGF‐1) is a secretory protein primarily synthesized by the liver and is composed of 70 amino acids. IGF‐1 has multiple important biological activities.^1^ The protein binds to its receptor, the insulin‐like growth factor receptor (IGF‐IR), activating IGF‐IR. This results in IGF‐IR auto‐phosphorylation, which in turn leads to phosphorylation of the insulin receptor substrate (IRS), and the tyrosine‐phosphorylated IRS‐1 initiates multiple signal cascade pathways, which taken together, participate in the growth and development of the body, regulate cell survival, proliferation, differentiation, apoptosis and necrosis.^2^

Since the discovery of IGF‐1s by Salmon and Daughaday in 1957, there has been increasing attention and research in this area.^3^ IGF‐1 belongs to a class of multi‐peptide growth factors involved in important roles in cell proliferation, differentiation and individual growth. Among them, IGF‐1 constitutes an important cytokine associated with the differentiation, proliferation and maturation of body tissues. IGF‐1 is also referred to as the growth‐promoting factor.^4^ This peptide‐protein is similar to insulin in molecular structure. Recent research evidence shows that IGF‐1 has significant biological effects in the intestines. Notably, IGF‐1 activates the PI3‐K pathway, promoting cell survival, and protects intestinal cells from ROS‐induced apoptosis.^5^ Besides, IGF‐1 protects intestinal epithelial cells from oxidative stress‐induced apoptosis.^6^ Further research evidence reveals that IGF‐1 plays a crucial role in the reconstitution of intestinal epithelial integrity.^7^ Studies show that the biological function of IGF is closely related to its cell behaviour. Traditionally, IGF‐1 was only thought to interact with its receptor on the cell membrane to activate intracellular signalling pathways in executing its functions. However, numerous studies indicate that IGF‐1 still has a vital biological activity after internalization into the nucleus.^8^, ^9^ Therefore, there is a need to explore the relationship between the cell behaviour of IGF‐1 and its biological function.

Although studies have shown that IGF‐1 has a critical biological effect in the intestine, there is lacking literature on the cellular behaviour and potential biological activity of IGF‐1/IGF1‐R in intestinal cells. Therefore, in the current study, we examined the biological characteristics and potential biological activities of IGF in intestinal cell models in detail. We established that in addition to the internalization IGF‐1 into the intestinal cells, it is also transported into the nucleus to play the corresponding biological role. Herein, we lay the foundation for detailed studies on the effect of IGF‐1/IGF‐1R on intestinal cells. At the same time, we show that nuclear‐localized IGF‐1R has the important biological activities.

## 2 MATERIALS AND METHODS

### 2.1 Materials

The serum and culture media were obtained from HyClone. Plasma Cell Fractionation Kit was purchased from Invent Biotechnologies Inc (Eden Prairie, MN, USA). Bovine serum albumin (BSA) and RIPA lysate were bought from Beyotime. We purchased horseradish peroxidase‐labelled goat anti‐rabbit IgG from BOSTER Biological Technology co. ltd. Antibodies against IGF‐1Rβ, EEA1, Rab5, Rab7, Rab9, Rab11, clathrin, caveolin and Nup358 were bought from Abcam. Anti‐total AKT and Anti‐p‐AKT, anti‐ERK1/2 and anti‐p‐ERK1/2, anti‐p‐IGF‐I Receptor β, and anti‐IGF‐1β were purchased from CST technology. The secondary antibody for the IF experiment, Alexa‐488‐conjugated goat anti‐mouse, was purchased from Molecular Probes (Life Technologies, Stockholm, Sweden). All the reagents were purchased from Sigma without special instructions.

### 2.2 Cell culture

The human intestinal epithelial cell line (hIEC6) (ATCC) and rat intestinal epithelial cell line (IEC6) (ATCC) were cultured in DMEM supplemented with 10% foetal bovine serum.

### 2.3 Immunoprecipitation

After the cells were treated with IGF‐1 at 30 min, the cells were washed twice using cold PBS solution, collected and then centrifuged at 400g for 3 min. The cells were re‐suspended in 500μl of cell lysate, frozen and thawed 3 times using liquid nitrogen. After that, we centrifuged the cells at 13 800 × g for 15min to remove the cell debris. The cell supernatant was mixed with agarose‐conjugated antibody incubated at 4°C for 4 h and then centrifuged at 400g for 5 min. The supernatant was discarded, and the pellet was washed for three times using RIPA. Subsequently, immunoprecipitation and Western blot analysis was performed as described below.

### 2.4 Western blot

The cells were extracted using the RIPA lysate buffer (300μl RIPA + 4μl PMSF). Total protein was determined using the BCA method. The protein samples were subjected to SDS‐polyacrylamide gel electrophoresis. We transferred the electrophoresed protein to a PVDF membrane and blocked with 3% BSA blocking solution, followed by overnight incubation at 4°C. After that, we incubated the membrane with the indicated antibodies at room temperature for 3 h. The membrane was then washed thrice (5 min/time) using TBS and incubated with fluorescently labelled secondary antibody) at room temperature for 2 h. Subsequently, the membranes were washed three times (5 min/time) using TBS and detected the blots using the ECL Plus detection system. We used the Quantity One image analysis software (Bio‐Rad) to calculate the gray value (densitometer unit, DU) of each group of protein bands.

### 2.5 EMSA (NIRF‐EMSA)

After stimulating the cells under the indicated conditions, they were washed twice using PBS. We extracted nuclear proteins using the Nucleoprotein Protein Extraction kit according to the instructions of the manufacturer. The extracted nucleoproteins were stored at −80°C for the subsequent experiments. The nuclear proteins were mixed with 5μl DNA binding buffer and incubated at room temperature for 60 min. After that, we added the fluorescent molecule‐labelled dsDNA probe (5’CATTTCCCGTAAATC3’ and 5’GATTTACGGGAAATG3’^10^), specific competition dsDNA or non‐specific competition dsDNA and incubated at room temperature for another 60 min. Then, the mixture was subjected to non‐denaturing electrophoresis, and the Odyssey infrared gel imager (Li‐Cor) was used to scan gel.

### 2.6 Indirect immunofluorescence assay (IFA)

After treating the cells with IGF‐1, the cells were fixed with a fixing solution (formaldehyde and acetone at a ratio of 1:1) and incubated at room temperature for 10 min. We then washed the cells thrice using PBS, followed by blocking at 37°C with 1% BSA for 1 h. Subsequently, the cells were washed three times in PBS, and then the primary antibody was added, followed by incubation at 4°C overnight. After three washes, the secondary antibody was added and the cells were incubated for 1 h at 37°C. After that, we washed the cells thrice and then stained the nuclei with DAPI. The cells were observed using a confocal microscope (Olympus FV3000).

### 2.7 ELISA assay

The cells were first treated using the IGF‐1 at the indicated time points. Then, we extracted the cytoplasm and nuclei proteins at the indicated time points. Next, ELISA assay was performed according to the instructions of the ELISA kit.

### 2.8 Cell proliferation assay

We assayed for cell proliferation using the CCK‐8 kit as per the instructions of the manufacturer. Cell suspensions (100 μl/well) were added into the 96‐well plate and incubated for 24h at 37℃. After washing the cells, IGF‐1 was added and incubated for 24h at 37℃. Then, we washed the cells and added 10μl of the CCK solution, followed by incubation for 2h at 37℃. The ELISA reader (Bio‐Rad, USA) was used to read the OD values at an absorbance of 450 nm.

### 2.9 Chromatin immunoprecipitation

The cells were treated with IGF‐1 for 30 min. Subsequently, we collected 2 × 10^7^ cells and added them into 20 ml PBS. The cells were fixed using formaldehyde for 15min. Then, we treated the cells with 1 mol/L of glycine for 5min, followed by washing twice in PBS. After that, the cells were centrifuged for 5min at 1000 rpm and then lysed using 2ml of the SDS lysate buffer. The chromatin DNA was sheared into ~ 1000bp fragments through ultrasonic treatment. Subsequently, the corresponding antibodies were used for immunoprecipitation using the Millipore's Chromatin Immunoprecipitation Kit as per the manufacturer's protocol. A PCR assay was performed to identify the DNA fragments obtained from the chromatin immunoprecipitation (ChIP).

### 2.10 Cell cycle analysis by flow cytometry

The cells were prepared as a single cell suspension and centrifuged at 800‐1000 rpm for 5min. We discarded the supernatant and then added 1ml PBS, followed by centrifugation for 5 min. Then, the supernatant was discarded. The cells were treated with 70% alcohol at 4°C for 30 min. After centrifugation, the supernatant was discarded. The cell pellets were then treated with RNase A in PBS (20 ug/ml) and incubated at 37°C for 30 min. Subsequently, we incubated the cell samples with PI for 30min at room temperature and protected from light. The cell samples were mixed and filtered through a 300‐mesh sieve, and then flow cytometry was performed with a FACScan instrument and the CellQuest software program (Becton Dickinson).

### 2.11 Alive measurement of cell bio‐behaviours by Cell‐IQ

The cells were seeded into a 24‐well cell culture plate (Costar) at 40,000 cells/well, and the corresponding stimuli performed individually and in combination according to the experimental design. The culture plates were placed in a special incubator for Cell‐IQ (37°C, 5% CO_2_) during the recording, and the real‐time tracking recording was performed using the Cell‐IQ imaging software. Subsequently, the Cell‐IQ analysis software was used to analyse the corresponding parameters.

### 2.12 Intracellular signalling

The cells were stimulated using IGF‐1 at the indicated time and dose points, and then intracellular signals were analysed using Western blot.

### 2.13 Duolink in situ PLA

The ICE6 cells were cultured on coverslips and fixed using 4% paraformaldehyde for 15min. After that, we permeabilized the cells using 0.2% Triton for 20min. The cells were then washed and blocked using 5% bovine serum albumin and 5% donkey serum for 1h. The cells were then incubated with primary antibodies overnight at room temperature for 1h. We then performed the in situ proximity ligation assay (PLA) using the Duolink in situ kit (Olink Bioscience, Uppsala Sweden), as per the manufacturer's instructions. CLSM was used to analyse all the IF experiments. The images were analysed in Image J software (developed by NIH).

### 2.14 Cell transfection

The IEC6 and HIEC6 cells were cultured in the DMEM medium supplemented with 10% FBS. We used lipofectamine 3000 (Invitrogen, Carlsbad, CA, USA) to transfect cells with siRNAs against importin‐β, Nup358, syntaxin6 and Rab5 (Dharmacon, Pittsburgh, PA, USA) and siRNA control (Dharmacon, Pittsburgh, PA, USA). Through Western blot, we determined the knock‐down efficiency and specificity of siRNA.

### 2.15 Fluorescence resonance energy transfer (FRET)

We performed the FRET experiment, as described previously.^7^ Briefly, the IEC6 cells were transfected with pEGFP‐IGF‐1R (donor) using lipofectamine 3000 as per the manufacturer's instructions. After transfection for 48h, the cells were cultured without serum for 10 h and then treated with IGF‐1 (50 ng/ml) for 30 min. We then washed the cells and fixed them with 4% paraformaldehyde overnight. The cells were washed thrice with PBS, followed by incubation with 0.05% Triton X‐100 for 15min. Subsequently, we permeabilized the cells and then blocked using 3% bovine serum albumin for 1h. After that, the cells were washed three times, then incubated with the primary antibodies (such as anti‐tubulin) for 1 h, and then incubated with Alexa Fluor 555 labelled secondary antibodies (acceptors) for 1h. The cells were analysed using the Olympus FV3000 confocal microscope. Regarding the FRET experiments, three channels were set up: donor (GFP), acceptor (Alexa Fluor 555) and FRET channels. The donor channel had an excitation set of 488 nm and an emission set of 495‐525nm, and the acceptor channel had an excitation set of 561nm and an emission set of 575‐635nm. In contrast, the FRET channel had a donor excitation of 488nm and an acceptor emission set of 575‐635 nm, which transferred the image from the acceptor.

### 2.16 Statistical analysis

All the data herein are indicated as mean ± SD. The data were analysed using a one‐way variance analysis in SPSS V23.0. A p‐value of <0.05 indicated statistical significance.

## 3 RESULTS

### 3.1 IGF‐1Rs are expressed on IEC6 cell

**FIGURE 1:**
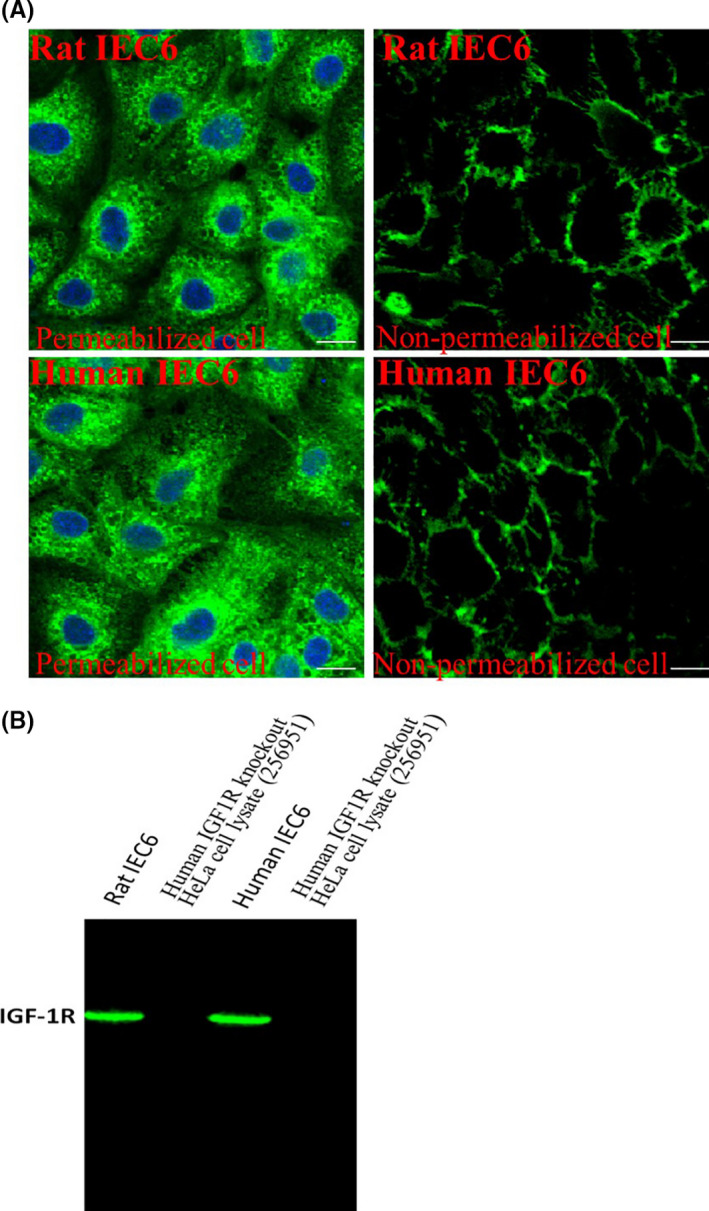
A, The expression of IGF‐1R as determined by IF in the permeabilized and non‐permeabilized cells. The cells were treated as described as in Materials and Methods section. B, The expression level of IGF‐1R was determined using Western blot

We analysed the expression and distribution of IGF‐1R on intestinal cells via IFA. Our results showed that IGF‐1Rs were expressed in the cell membrane as well as in the cytoplasm (Figure 1A). Additionally, the Western blot analysis results indicated that IGF‐1R proteins were expressed in the rat IEC6 and human IEC6 cells (Figure 1B). Furthermore, we also evaluated the expression of IGF‐1R by flow cytometry (Figure S1).

### 3.2 Internalization of IGF‐1 in rat IEC6 and human IEC6 cells

**FIGURE 2:**
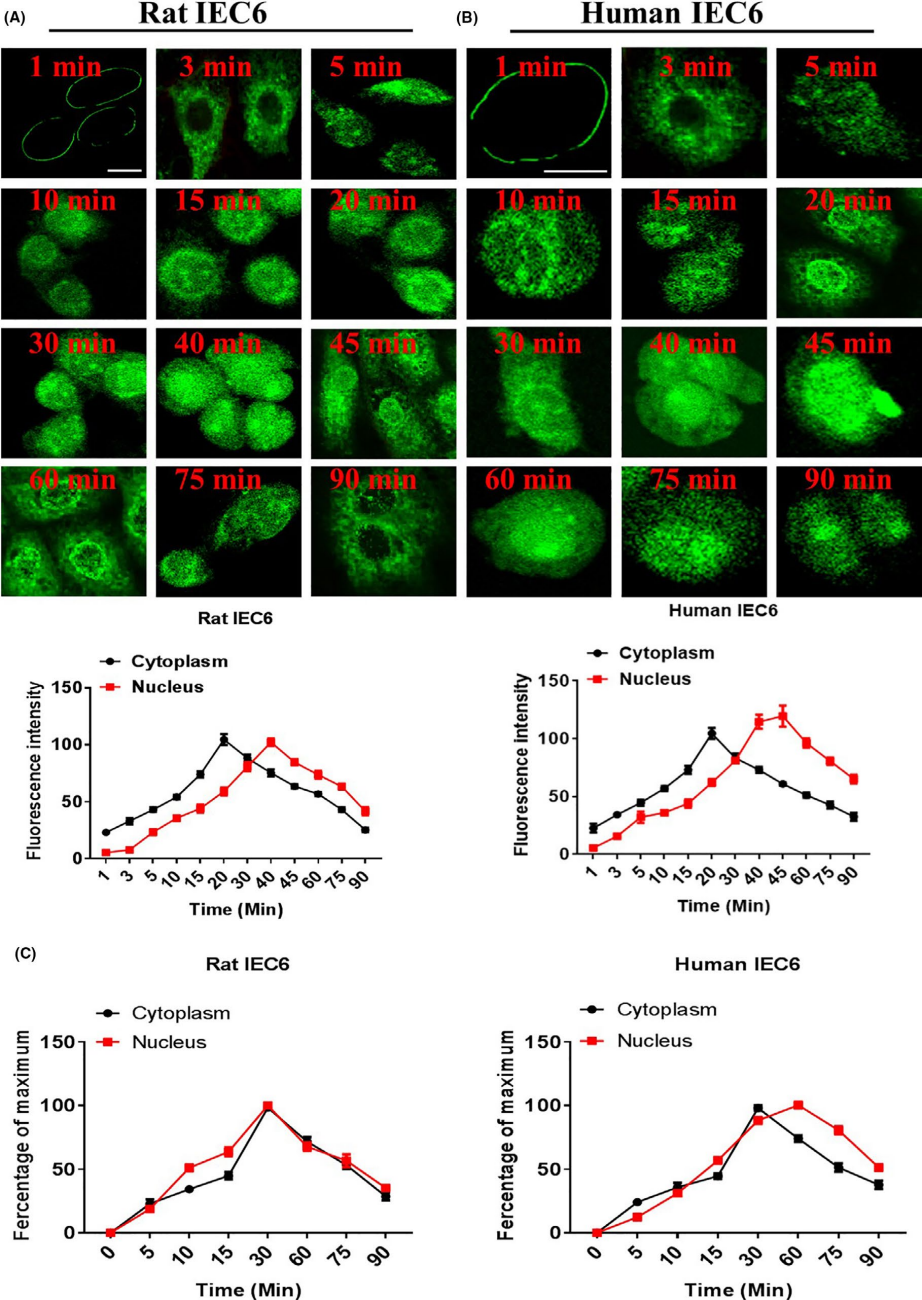
Internalization kinetics of IGF‐1 based on cell models of rat IEC6 (A) and hIEC6 (B). The cells were serum‐starved for 6 h, washed and incubated with Alexa‐488‐IGF‐1 for different durations.C, Measurement of IGF‐1 by ELISA assays. The data shown are the mean ± SD of three independent experiments. The pictures shown are representative of three independent experiments

We first studied the internalization kinetics of IGF‐1 on the cell models of IEC6 and hIEC6. The cells were incubated with IGF‐1 at different time points and then analysed using confocal microscopy. In the rat IEC6 cell model, we found that Alexa‐488‐IGF‐1 was internalized into cells in a time‐dependent manner (Figure 2A; Figure S2). At 0‐1 minutes, IGF‐1 was localized on the cell membrane and cytoplasm. The levels of IGF‐1 internalized into the cytoplasm gradually increased with the increasing incubation time. At 30‐45 min, the IGF‐1 intracellular fluorescence signal reached its peak. Subsequently, the fluorescence signal of IGF‐1 gradually weakened at 60 min. Moreover, in the human IEC6 cell model (Figure 2B; Figure S3), similar to the rat IEC6 cell model, at 1 min, the IGF‐1 was mainly localized on the cell membrane, with little or no fluorescence in the cytoplasm. Then the cytoplasm fluorescence increased with increasing incubation time. The fluorescence gradually increased and reached its maximum at the incubation time of 30‐60 min and then began to decline slightly. Notably, we also found that IGF‐1 was internalized into the nucleus in both the intestinal cell lines in a time‐dependent manner. Furthermore, we examined the subcellular components via ELISA and found consistent results with the confocal analysis findings (Figure 2C).

### 3.3 Intracellular trafficking of IGF‐1R is induced by IGF‐1 in two intestinal cells

**FIGURE 3:**
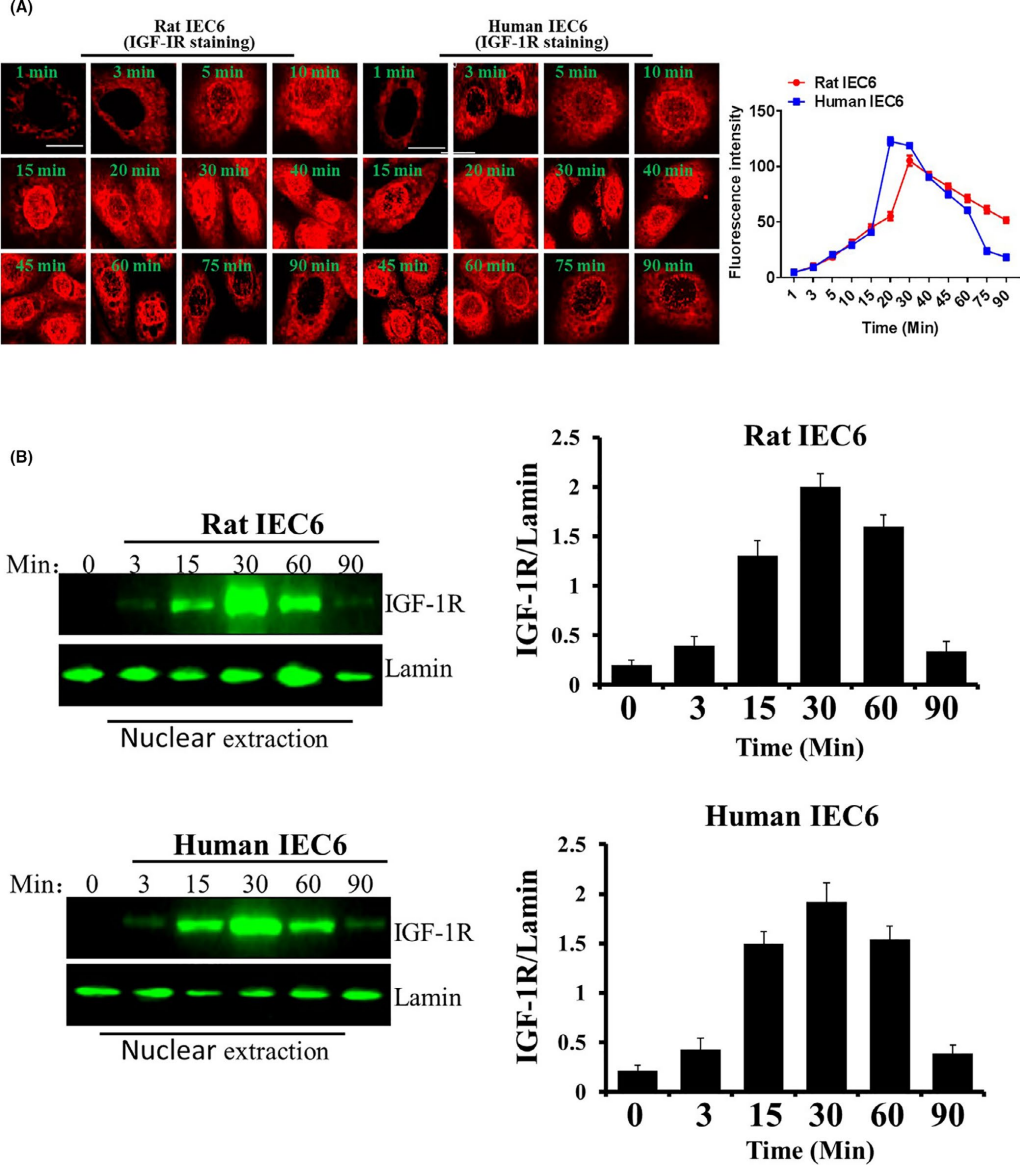
Nuclear localization of IGF‐1R induced by IGF‐1. A, The cells were treated with IGF‐1 for the indicated durations. The kinetics of IGF‐1R nuclear localization was analysed by CLSM. The pictures shown are representative of three independent experiments. B, Kinetics of nuclear localization of IGF‐1R was examined by Western blot. The data shown are the mean ± SD of three independent experiments

Research evidence shows that IGF‐1R executes its biological role through two pathways, namely the cytoplasmic pathway (traditional) and the nuclear pathway [8]. Herein, we analysed the kinetics of IGF‐1R nuclear localization induced by IGF‐1. Our results showed that IGF induced the entry of IGF‐1R into the cell nuclei in the two intestinal cell lines in a time‐dependent manner (Figure 3A; Figure S4). Nuclear‐localized IGF‐1R was initially detected 5 minutes after IGF‐1 treatment and reached its maximum at the incubation time of 45‐60 minutes. These results were consistent with the findings of the kinetics of nuclear localization of IGF‐1R analysis through Western blot analyses (Figure 3B). These findings suggest that IGF‐1R has important roles in the intestinal cell nuclei.

### 3.4 Endocytosis of IGF‐1/IGF‐1R is mediated by clathrin and caveolin

**FIGURE 4:**
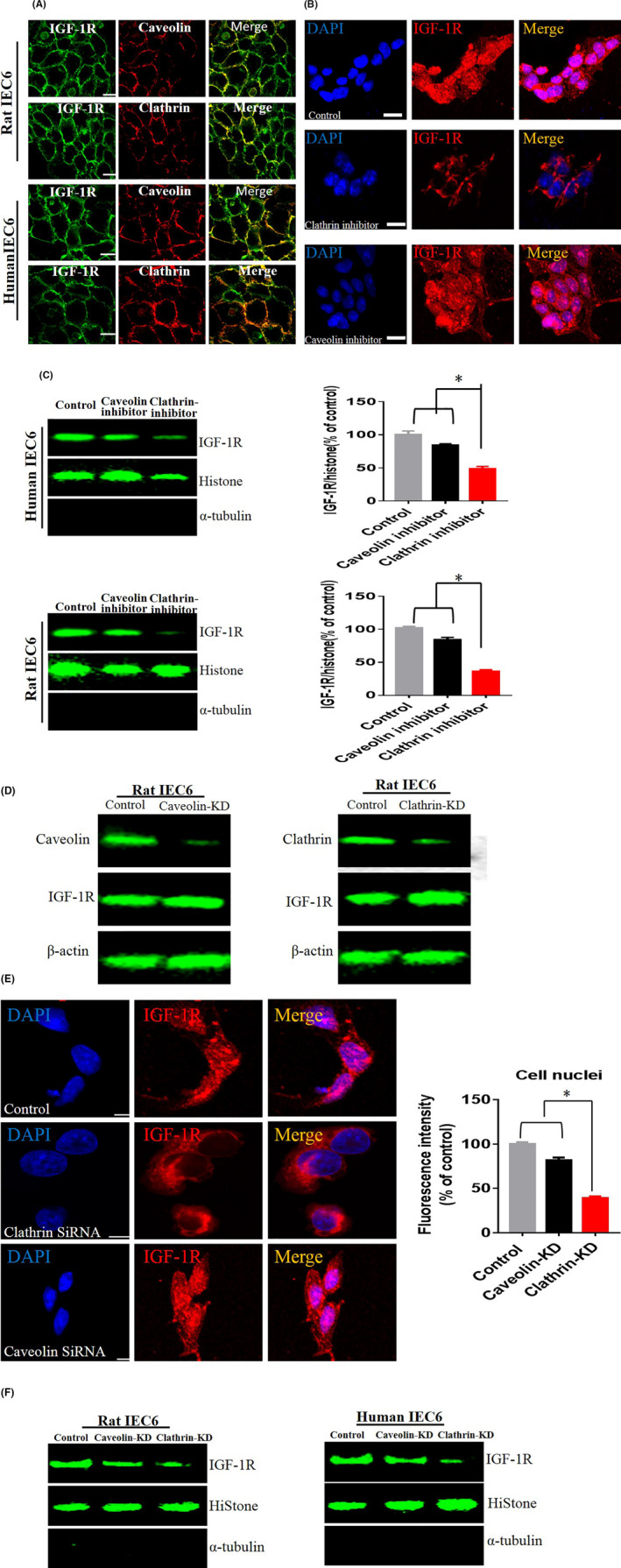
A, Co‐localization analysis of clathrin/IGF‐1R and caveolin/IGF‐1R by IF. B and C, Detecting the nuclear localization of IGF‐1R by inhibiting clathrin and caveolin‐mediated endocytosis. The cells were treated with caveolin or clathrin inhibitor (genistein, Pitstop) and then incubated with IGF‐1 for 30 min. D, Knock‐down clathrin and caveolin through siRNA technology. E and F, Internalization and nuclear localization of IGF‐1/IGF‐1R were assessed by IF or Western blot. Knock‐down of caveolin or clathrin expression in IEC6 cells was performed by siRNA. The signal from control cells was considered 100%. The figures are representative of at least three independent experiments. An asterisk (*) indicates a significant difference

We first established the nuclear localization of IGF‐1/IGF‐1R in intestinal cells. Next, we studied the mechanism involved in the nuclear localization of the IGF‐1/IGF‐1R. The endocytosis of IGF‐1/IGF‐1R constitutes the initial step of their nuclear transport. Previous research findings indicated that the endocytic pathway of the same cytokine/receptor is different in different types of cells. In intestinal cells, the endocytic pathway of the IGF‐1/IGF‐1R from the plasma membrane to the cytoplasm remains unclear. At present, three main types of endocytic pathways for cytokines and hormones have been described, namely clathrin‐dependent endocytosis, the caveolin‐dependent endocytic pathway and the non‐clathrin‐ and caveolin‐dependent pathway.^6^ Herein, we performed co‐localization analysis to explore the endocytic pathway of the IGF‐1/IGF‐1R, and we detected the co‐localization signals of caveolin/IGF‐1R and clathrin/IGF‐1R, suggesting that clathrin and caveolin are involved in the endocytosis of IGF‐1/IGF‐1R (Figure 4A). To validate which endocytic pathway (clathrin‐mediated endocytosis or caveolin‐mediated endocytosis) is involved in the nuclear localization of IGF‐1/IGF‐1R, caveolin‐specific inhibitor or clathrin‐specific inhibitor was used to block caveolin‐mediated or clathrin‐mediated endocytosis, respectively, and the results showed that clathrin‐specific inhibitor markedly reduced the nuclear localization of IGF‐1R (Figure 4B and C), suggesting that clathrin‐mediated endocytosis may play a major role in the nuclear localization of IGF‐1R. We then confirmed these findings by knocking down caveolin and clathrin through siRNA technology, respectively. As shown Figure 4D, the expressions of caveolin and clathrin were markedly reduced. However, there was no remarkable decrease in the protein levels of IGF‐1R, and the knock‐down of clathrin (but not caveolin) markedly reduced the nuclear localization of IGF‐1R (Figure 4E and F). These findings suggest that clathrin may play a more important role in the process of the IGF‐1R’s nuclear localization, although both clathrin‐ and caveolin‐mediated endocytosis are all involved in IGF‐1R’s internalization.

### 3.5 The internalized IGF‐1R enters different types of endosomes

**FIGURE 5:**
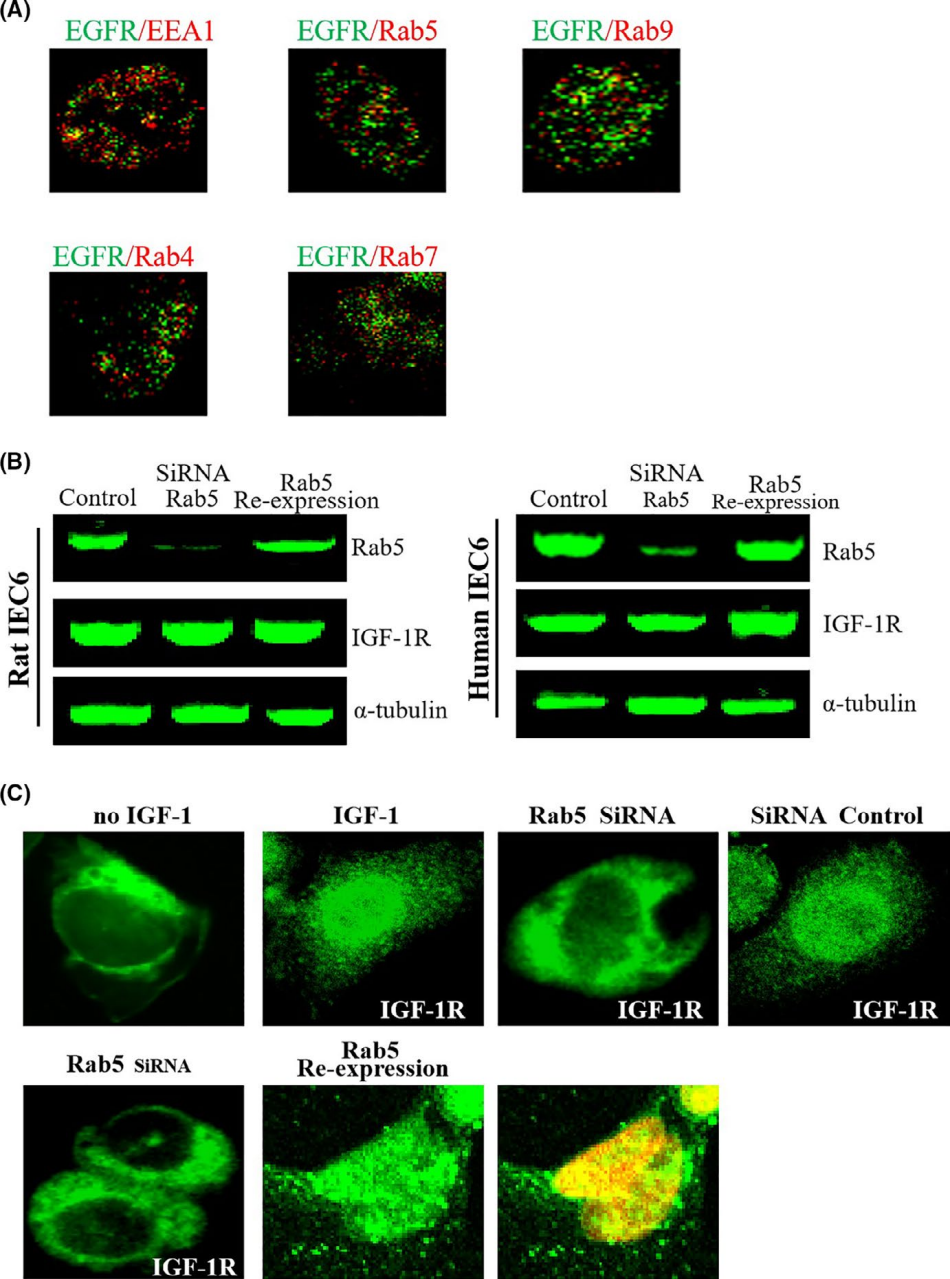
A, The internalized IGF‐1R enters to different types of endosomes under IGF‐1 stimulation. Cells were incubated with IGF‐1 for 30 min and then with the indicated antibodies. Co‐localization signal was detected by CLSM. Yellow in the merged pictures indicates colocalization. Scale bar, 10 mm. B, Rab5 is required for nuclear localization of IGF‐1/IGF‐1R. C, Inhibition of Rab5 restored nuclear localization of IGF/IGF‐1R. The figures are representative of at least three independent experiments

We further analysed which types of endosomes the IGF/IGF1R proteins enter. Different endosomes play different roles in the trafficking of cargo molecules and transport different cargo molecules to distinct destinations.^11^ In the current study, we analysed the co‐localization signal of IGF‐1R and EEA1 (early endosome marker). In our results, we detected the co‐localization signals of IGF‐1R and EEA1, indicating the successful entry of IGF‐1R into the early endosome (Figure 5A; Figure S5) consistent with the findings of previous studies.^8^ Endosomes are classified further based on Rab GTPases.^11^ Early endosomes are rich in Rab5. The recycling endosome is rich in Rab4 as well as Rab11 (which mediate the cargo molecules to transport to the plasma membrane), whereas Rab7 and Rab9 are abundant in the late endosomes (which mediate cargo molecules targeted for lysosomal degradation).^12^ Herein, we perform the co‐localization experiments to explore which type of endosomes the IGF‐1R complex enters, and the results showed that IGF‐1R entered the endosomes rich in Rab4, Rab5 and Rab7, explaining why IGF‐1R showed different cytoplasmic localization.

To study further the role of Rab5 in the nuclear localization process of IGF‐1R, we silenced Rab5 through siRNA. We found that the nuclear localization of IGF‐1/IGF‐1R was significantly reduced, although the internalization and cytoplasmic localization of IGF‐1/IGF‐1R were not affected, suggesting that Rab5 participates in the nuclear localization of IGF‐1/IGF‐1R (Figure 5B and C). More results indicated that microtubules are involved in the cytoplasmic transport of IGF‐1R (please see 3.7 and 3.8 section). Additionally, we found that the co‐location of IGF‐1R and microtubules is significantly reduced by FERT analysis, suggesting that Rab5‐positive endosomes loaded with cargo molecules (IGF‐1/IGF‐1R) are dependent on microtubule transport. These findings indicated that IGF‐1/IGF‐1R entering into the Rab5‐positive endosomes are essential for nuclear localization of IGF‐1/IGF‐1R. To confirm further these findings, we rescued the expression of Rab5 by transfection. The results showed that the nuclear localization of IGF‐1R was significantly restored (Figure 5C).

### 3.6 Tyrosine phosphorylation of IGF‐1R is required for nuclear localization of multiple IGF‐1R

**FIGURE 6:**
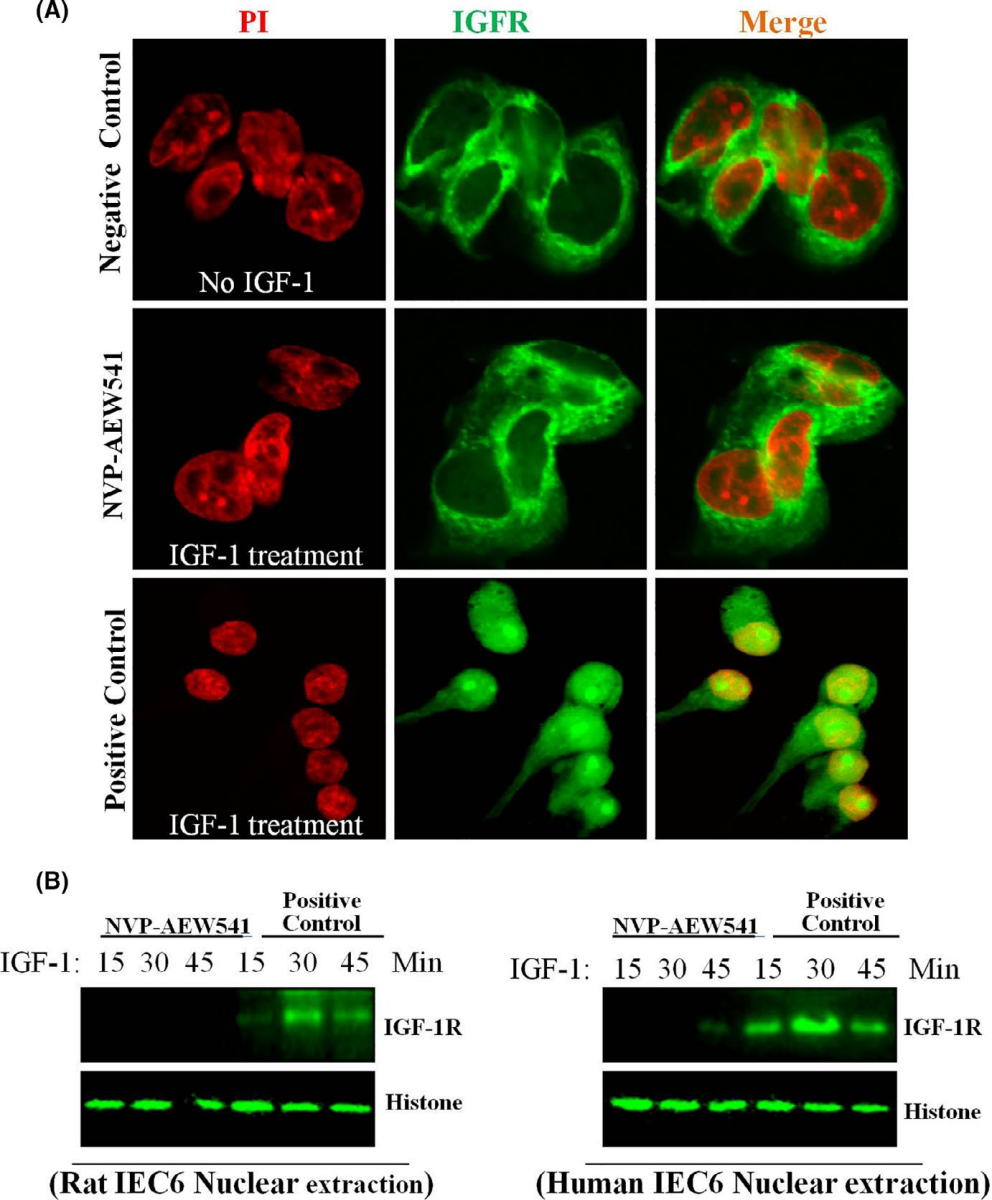
Tyrosine phosphorylation of IGF‐1R is required for nuclear localization of multiple IGF‐1R. Cells were treated with NVP‐AEW541and then analysed by CLSM. The pictures shown are representative of three independent experiments

Next, we determined whether the nuclear localization of IGF‐1R is associated with tyrosine phosphorylation of IGF‐1R. We used NVP‐AEW541 (IC50 value of 0.15 μM) to treat the cells, which is a highly selective inhibition of IGF‐1R phosphorylation through IGF‐1R kinase activity (Figure 6; Figure S6). We found that NVP‐AEW541 blocked the nuclear localization of IGF‐1R. This finding suggests that IGF‐1R phosphorylation is essential in the nuclear transport of IGF‐1R.

### 3.7 Golgi apparatus is an important site for nuclear localization of IGF‐1R

**FIGURE 7:**
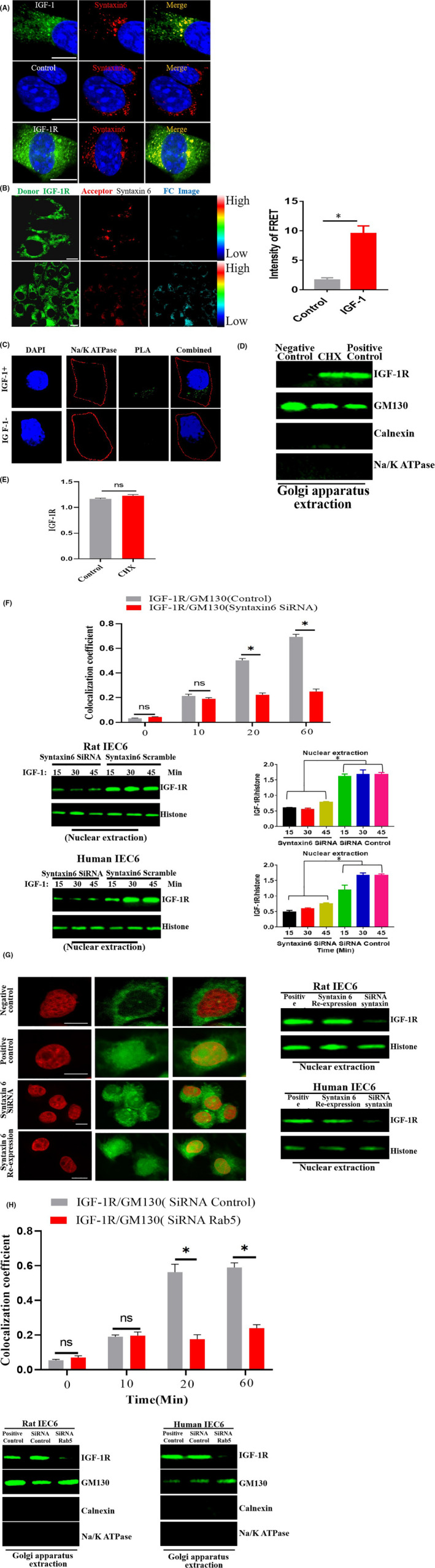
A, Analysis of EGF‐1R and syntaxin6 colocalization. Cells were treated with IGF‐1 for 30 min and incubated with the indicated primary antibodies, and followed by staining with fluorescent‐labelled secondary antibody. B, FRET analysis was performed to examine the interaction between IGF‐1R and syntaxin6. Cells were serum starved for 6 h and then treated without or with IGF‐1 (50 ng/ml) for 30 min. IGF‐1R and syntaxin6 were labelled with primary antibodies and secondary fluorescein isothiocyanate (donor, green) and Alexa‐555 (acceptor; red) antibody. An Fc image was analysed by using the Olympus 3000 software. Scale bar, 20 nM. Quantitation of the FRET intensity is shown in the right. C, Analysis of the interaction between IGF‐1R and syntaxin6 using PLA. D, Co‐localization of IGF‐1R and GM130 (Golgi marker) by CLSM. E, IGF‐1R protein and mRNA levels were quantified by Western blot and RT‐PCR assays, respectively. F, Knock‐down of syntaxin6 decreased nuclear localization of IGF‐1R G, Inhibition of Rab5 restored the nuclear localization and transport of IGF‐1R by Golgi. H, Rab‐5‐positive endosomes transport IGF‐1R to Golgi apparatus. The pictures shown are representative of three independent experiments. NS: Not Significant; An asterisk (*) indicates a significant difference

After IGF‐1 treatment, the co‐localization signal of IGF‐1R and syntaxin 6 (Golgi marker) was detected via laser confocal microscopy (Figure 7A). The FRET analysis results showed that IGF‐1 treatment led to energy transfer from fluorescein isothiocyanate‐labelled IGF‐1R (the donor) to Alexa Flour 555‐labelled syntaxin6 (the acceptor) (Figure 7B). Furthermore, the PLA findings showed that IGF‐1R was localized in the Golgi apparatus (Figure 7C). Similarly, previous studies also reported that IGF‐1R is localized in the Golgi apparatus in other types of cells.^13^ To rule out the possibility that IGF‐1 treatment induces IGF‐1R synthesis in the ER and post‐translational modification in the Golgi, we used the cycloheximide (CHX) to block protein synthesis. Our results revealed that IGF‐1 enhanced the IGF‐1R localization to Golgi apparatus (Figure 7D). This was supported further by the fact that mRNA levels did not change markedly following IGF‐1 stimulation (Figure 7E). Next, we explored whether the Golgi‐localized syntaxin6 participates in the nuclear localization process of IGF‐1/IGF‐1R. Using siRNA to silence the syntaxin6, we found that the nuclear localization of IGF‐1R, as well as the Golgi localization of IGF‐1R, was reduced (Figure 7F). Therefore, these results suggest that the Golgi apparatus plays a critical role in the nuclear transfer of IGF‐1R. To confirm further the role of syntaxin6 in the nuclear transport of IGF‐1R, we first disrupted the expression of syntaxin6 through a shRNA against its 3’‐UTR and then determined the levels of nuclear IGF‐1R after syntaxin6 was re‐expressed. The results indicated that the nuclear localization of IGF‐1/IGF‐1R was restored (Figure 7G). Altogether, these results show that syntaxin6‐mediated Golgi translocation of IGF‐1R is important in the nuclear translocation of IGF‐1R.

Next, we examined whether the Rab5‐positive endosomes are involved in the transport of IGF‐1R to the Golgi apparatus. Our results revealed that knocking down Rab‐5 reduced the co‐localization of IGF‐1R and syntaxin6. We further used the Golgi marker (GM130) to validate our findings. The results showed that the co‐localization of both IGF‐1R and GM130 were reduced (Figure 7H). This indicates that the Rab‐5 positive endosomes are responsible for the transport of IGF‐1R to the Golgi apparatus, suggesting that IGF‐1R entering the Rab‐5‐positive endosomes is a prerequisite for the entry of IGF‐1R into Golgi apparatus.

### 3.8 Microtubules participated in the nuclear localization of IGF‐1R in intestinal cells

**FIGURE 8:**
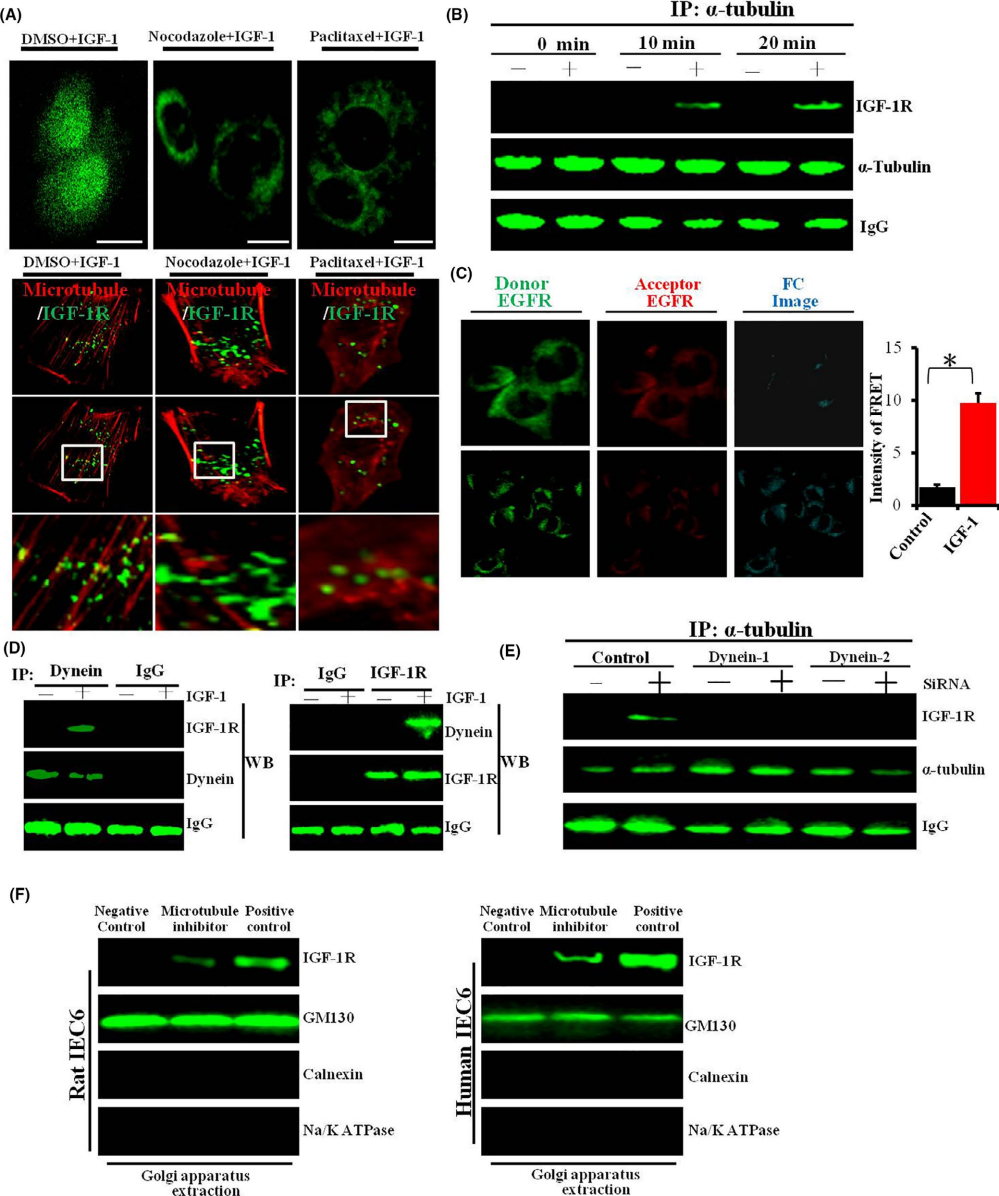
A, Nuclear localization of IGF‐1/IGF‐1R is inhibited. Cells were treated with inhibitors of microtubule formation (nocodazole or paclitaxel), labelled with the indicated antibodies and secondary antibody and examined by CLSM. B, Analysis of the interaction between IGF‐1R and tubulin with IP–WB assays. C, Analysis of the interaction between IGF‐1R and tubulin by FRET assay. Cells were serum‐starved for 6 h and then treated without or with IGF‐1 (50 ng/ml) for 30 min. IGF‐1R and tubulin were labelled with a primary antibodies and secondary fluorescein isothiocyanate (donor, green) and Alexa‐555 (acceptor; red) antibody. An Fc image was analysed using the Olympus 3000 software. Scale bar, 20 nm. Quantitation of the FRET intensity is shown in the right. D, Analysis of the interaction between IGF‐1R and dynein by IP–WB. E, The association of IGF‐1R with a‐tubulin was decreased following knock‐down of dynein by siRNAs. F, Analysis of Golgi localization of IGF‐1 after inhibition of microtubules as revealed by Western blot. The pictures shown are representative of three independent experiments

We used the inhibitors of microtubule formation (nocodazole and paclitaxel [6]) to establish whether microtubules are involved in the nuclear localization of IGF‐1R. We found that IGF‐1R is mainly concentrated in the cell membrane and cytoplasm domain (Figure 8A). This indicates that the microtubules are involved in the nuclear localization of IGF‐1/IGF‐1R. The nuclear localization of IGF‐1 was blocked in cells pretreated with nocodazole.

We further analysed the role of microtubules through Western blot and FERT assays. First, the IP‐Western blot results indicated that IGF‐1 enhanced the interaction between IGF‐1R and a‐tubulin (Figure 8B). Additionally, the FRET experiment showed that IGF‐1 treatment increased the energy transfer from EGFP‐fused IGF‐1R (the donor) to the Alexa Flour 555‐labelled microtubules (the acceptor) (Figure 8C). These results show that IGF‐1 induces IGF‐1R to localize around microtubules, suggesting that IGF‐1R moves along microtubules upon IGF‐1 stimulation.

The cytoplasmic dynein/dynactin complex are microtubule‐associated motors that drive cargo movement along the microtubules. Therefore, we further investigated whether IGF‐1 induces the association of IGF‐1R with dynein. The results of the IP–WB analyses indicted that IGF‐1 induced the association of IGF‐1R with dynein (Figure 8D). Notably, the knock‐down of dynein by siRNAs decreased the association of IGF‐1R with a‐tubulin (Figure 8E). These results suggest that dynein is involved in a microtubule‐dependent movement of IGF‐1R.

Besides, the inhibition of microtubules reduced the Golgi localization of IGF‐1R (Figure 8F), indicating that the Rab5‐positive endosomes that carry the cargo molecules (IGF‐1R) move on the microtubules. Similar findings have been reported previously.^14^

### 3.9 Nup358 is involved in the nuclear localization of IGF‐1R on intestinal cell models

**FIGURE 9:**
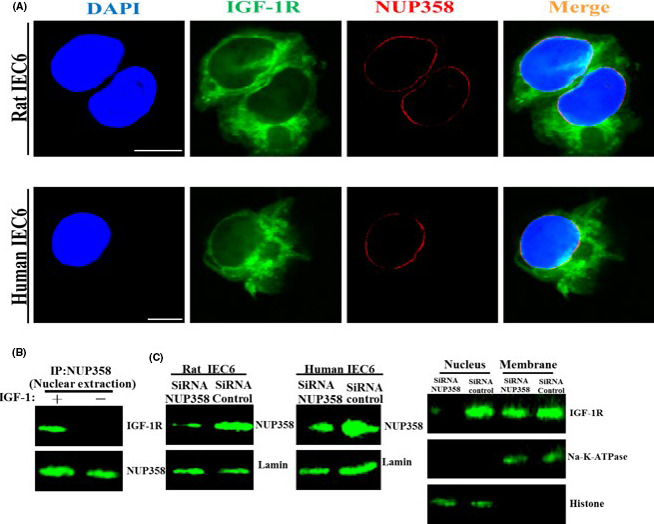
A, Analysis of the interaction between IGF‐1R and Nup358 by PLA assay. B, Analysis of the interaction between IGF‐1R and Nup358 by IP‐WB assay. C, Knock‐down of Nup358 decreased nuclear localization of IGF‐1R. The pictures shown are representative of three independent experiments

The last step of IGF‐1R involves the crossing of IGF‐1R through the nuclear membrane into the nucleus. The findings of previous studies showed that NUP358 (RanBP2) is involved in the nuclear localization of IGF‐1R in other cell types.^14^ Herein, we explored whether NUP358 is involved in the nuclear localization of IGF‐1R in intestinal cell models. The results of the colocalization analyses revealed that IGF‐1R can interact with Nup358 (Figure 9A). The IP‐WB results validated the association between these two proteins (Figure 9B). We further reduced the expression of Nup358 via siRNA and used CHX to inhibit the synthesis of new Nup358. The results showed that the nuclear localization of IGF‐1R was significantly reduced (Figure 9C), indicating that NUP‐358 is involved in the nuclear localization of IGF‐1R consistent with the findings of previous studies.^14^

### 3.10 Nuclear‐localized IGF‐1R increases nuclear retention of signalling molecule

**FIGURE 10:**
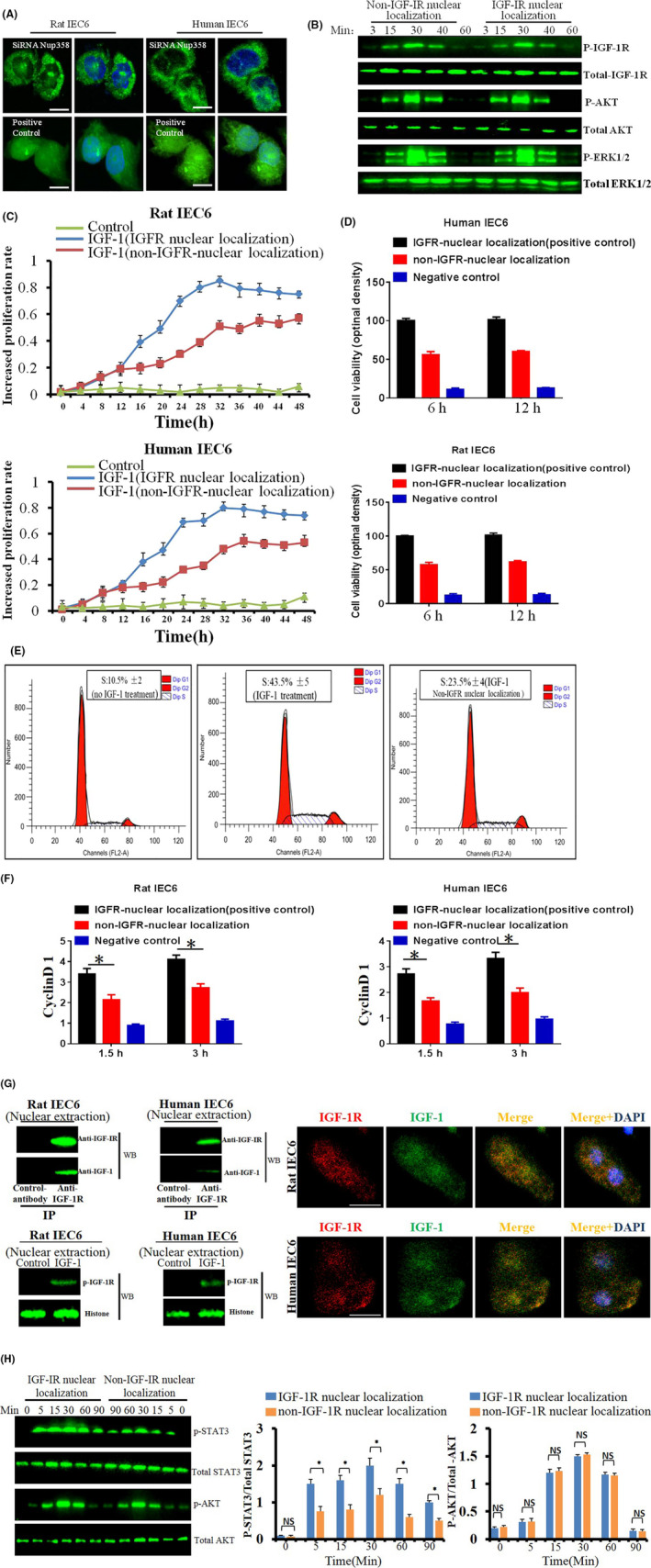
A, knock‐down of Nup‐358 decreased nuclear localization of IGF‐1R significantly as detected by CLSM. B, Analysis of IGF‐1R‐mediated signals at the indicated time points. C, IGF‐1 stimulation increased the proliferation rate of IEC6 and hIEC6 cells in normal group and in ‘non‐IGFR‐nuclear localization’ group. D, Analysis of cell proliferation by CCK8 kit. The data shown are the mean ± SD of three independent experiments. *P* <.05 was considered significant. E, The cell cycle was analysed by flow cytometry. F, Analysis of the nuclear localization of cyclin‐1 by IF. G, IGF‐1R formed dimers with IGF‐1 in the cell nuclei. H, IGF‐1‐induced STAT3 signalling in the cell nuclei was significantly prolonged compared to non‐nuclear‐localized IGF‐1R/IGF‐1R

Furthermore, we explored the potential role of nuclear localization of IGF‐1R. Therefore, we first had to establish a model of the non‐nuclear localization of IGF‐1R. We created this cell model by knocking down Nup358. Consequently, the nuclear localization of IGF‐1R was significantly reduced (Figure 10A), but its internalization and cytoplasmic localization of IGF‐1/IGF‐1R were not affected. Moreover, IGF‐1R‐mediated signalling was not significantly affected (Figure 10B; Figure [Link], [Link]).

We then analysed the change of cell bio‐behaviours using Cell‐IQ, and results showed that although IGF‐1 increased significantly, the proliferation rate in IEC6 and hIEC6 cells in the nuclear localization group was markedly higher compared with the non‐IGFR‐nuclear localization group under IGF‐1 stimulation. This suggests that the nuclear localization of IGF‐1R was associated with cell proliferation (Figure 10C). Additionally, the CCK8 results verified these findings (Figure 10D). Furthermore, we found that the nuclear‐localized IGF‐1R increased the cell cycle as well as the expression of cyclin‐D1 (Figure 10E and F).

Moreover, we found that the nuclear‐localized IGF‐1R formed a dimer with IGF‐1 (at least partially) (Figure 10G). The nuclear‐localized IGF‐1R was phosphorylated (Figure 10G), which prompted us to hypothesize that the biological activity of the nuclear‐localized IGF‐1/IGF‐1R is similar to the cell membrane‐localized IGF‐1/IGF‐1R. Therefore, we studied the relationship between IGF‐1/IGF‐1R and its mediating downstream signalling. Consequently, we found that compared with the non‐nuclear‐localized IGF‐1/IGF‐1R, the time course of the IGF‐1‐induced p‐STAT3 in the cell nuclei was significantly prolonged and increased. This implies that the nuclear localization time of the signal molecule is prolonged (Figure 10H). Furthermore, the EMSA results indicated that the transcription activity of nuclear STAT3 was elevated (Figure S8). These results suggested that the kinase activity of IGF‐1R is required to execute the biological function of IGF‐1R localized in the cell nuclei. In addition, we also have performed the chromatin immunoprecipitation assay to analyse whether IGF‐1R interacts with the transcription factors, and we found that IGF‐1R binds to the promoter of IGF‐1R and increases its expression consistent with previous findings, which was reported by previous work.^14^

In addition to establishing the cell model of non‐nuclear localization IGF‐1R by knocking down Nup‐358, we also establish a model of the non‐nuclear localization of IGF‐1R by knocking down Rab5 (as shown in Figure 5), and a series of research results also indicated that the nuclear‐localized IGF‐1R increases nuclear retention of STAT3, also prompting cell proliferation (Figure [Link], [Link], [Link], [Link], [Link], [Link], [Link]). This finding is consistent with the above‐mentioned results, which further confirms our findings.

## 4 DISCUSSION

In this study, the nuclear localization of IGF‐1R in intestinal cells was reported for the first time by us. Furthermore, we revealed the molecular mechanism by which IGF‐1R transports into cell nuclei and the potential biological role of the nuclear‐localized IGF‐1R. Although the phenomenon of IGF‐1R’s nuclear localization has been reported in different types of cells,^14^, ^15^, ^16^, ^17^, ^18^ the molecular mechanism by which IGF‐1R entering into cell nuclei is still unclear. In the current research, we have systematically analysed this scientific problem. In general, the nuclear transport of IGF‐1/IGF‐1R can be divided into three basic processes: 1) how the IGF‐1/IGF‐1R endocytosed into the cytoplasm from the cell membrane; 2) how the internalized IGF‐1/IGF‐1R is sorted in the cytoplasm; 3) How the cytoplasm‐localized IGF‐1/IGF‐1R passes through the nuclear pore. Several pathways are participated in the cell surface receptor endocytosis, including clathrin‐mediated, caveolin‐mediated and non‐clathrin‐ and non‐caveolin‐mediated pathways.^19^, ^20^, ^21^, ^22^, ^23^, ^24^, ^25^ Receptor endocytosis is the first step in nuclear transport of IGF‐1/IGF‐1R.^9^ In the current work, we showed that although both clathrin and caveolin are involved in the endocytosis of IGF‐1R, the clathrin‐mediated endocytosis plays a more vital role in the nuclear localization process of IGF‐1R, which is similar with the previous report.^20^

Endocytosis constitutes the initial step in the nuclear localization of IGF‐1R. The second important step is the cytoplasmic trafficking of IGF/IGF‐1R. The transport of macromolecular proteins in the cytoplasm requires the mediation of endosomes. Therefore, we identified the type of endosomes that IGF‐1R enters into, and co‐localization analyses showed that IGF‐1R enters different types of endosomes (EEA1, Rab5 and Rab7). Notably, we found that knocking down Rab5 suppresses the nuclear localization of IGF‐1R, and re‐expression of Rab5 restores the nuclear localization of IGF‐1R, suggesting that the Rab5‐positive endosomes are involved in the nuclear localization of IGF‐1R. Besides the endosomes, the cytoplasmic trafficking of IGF‐1R may additionally require cofactors. We found that microtubules and motor proteins (dynein/dynactin motor complex) were also involved in the intracellular transport of IGF‐1R. Packham et al have found that dynactin plays a vital role in the nuclear localization of IGF‐1R.^14^ In addition, Du et al also reported dynein/dynactin motor complex is involved in the nuclear localization of EGFR.^6^ Next, we tested whether cytoplasmic organelles (endoplasmic reticulum and Golgi apparatus) may also regulate nuclear localization of IGF‐1/IGF‐1R. In previous studies, it was found that cytoplasmic organelles may act as a ‘bus station’ and as an important ‘transit station’ in the nuclear transport of growth factor receptors.^11^, ^15^ The current study shows that Golgi apparatus may play an important role during nuclear transport of IGF‐1R, suggesting that Golgi apparatus is likely to be an important ‘transit station’ in the process of nuclear transport of IGF‐1R. In addition, in the initial few minutes after IGF‐1 stimulation, we observed a direct contact of IGF‐1‐labelled vesicular structures with the nuclear membrane (NM) (Figure S10). It seems that these vesicle‐like structures can dock on the nuclear envelope and then discharge their cargo (IGF‐1 and/or IGF‐1R) into the cell nuclei (termed as endosome‐nuclear membrane pathway). Of course, this is only our preliminary experimental observation, and more experiments are needed to prove this important scientific issue. Most recently, Lan et al observed that pGH‐labelled vesicle‐like structures could fuse with nuclear membrane and then pGH‐labelled vesicular structures were directly transported into the cell nuclei.^26^ In addition, the similar phenomenon has also been reported by other group.^27^ Recently, NAE (nucleus‐associated endosomes) model was proposed by some scientists. They postulate that there is a population of endosomes which directly targeted to nuclear envelope. These endosomes loaded with biological macromolecules could dock and fuse with the outer nuclear membrane (ONM), and then cargo molecule (such as EGFR) translocate into the inner nuclear membrane (INM) mediated by the nuclear pore complex (NPC), after which cargo molecule (such as EGFR) enter into cell nuclei.^28^ Of course, more work is required to prove this pathway (NAE), for example whether organelles are involved in this process.

**FIGURE 11:**
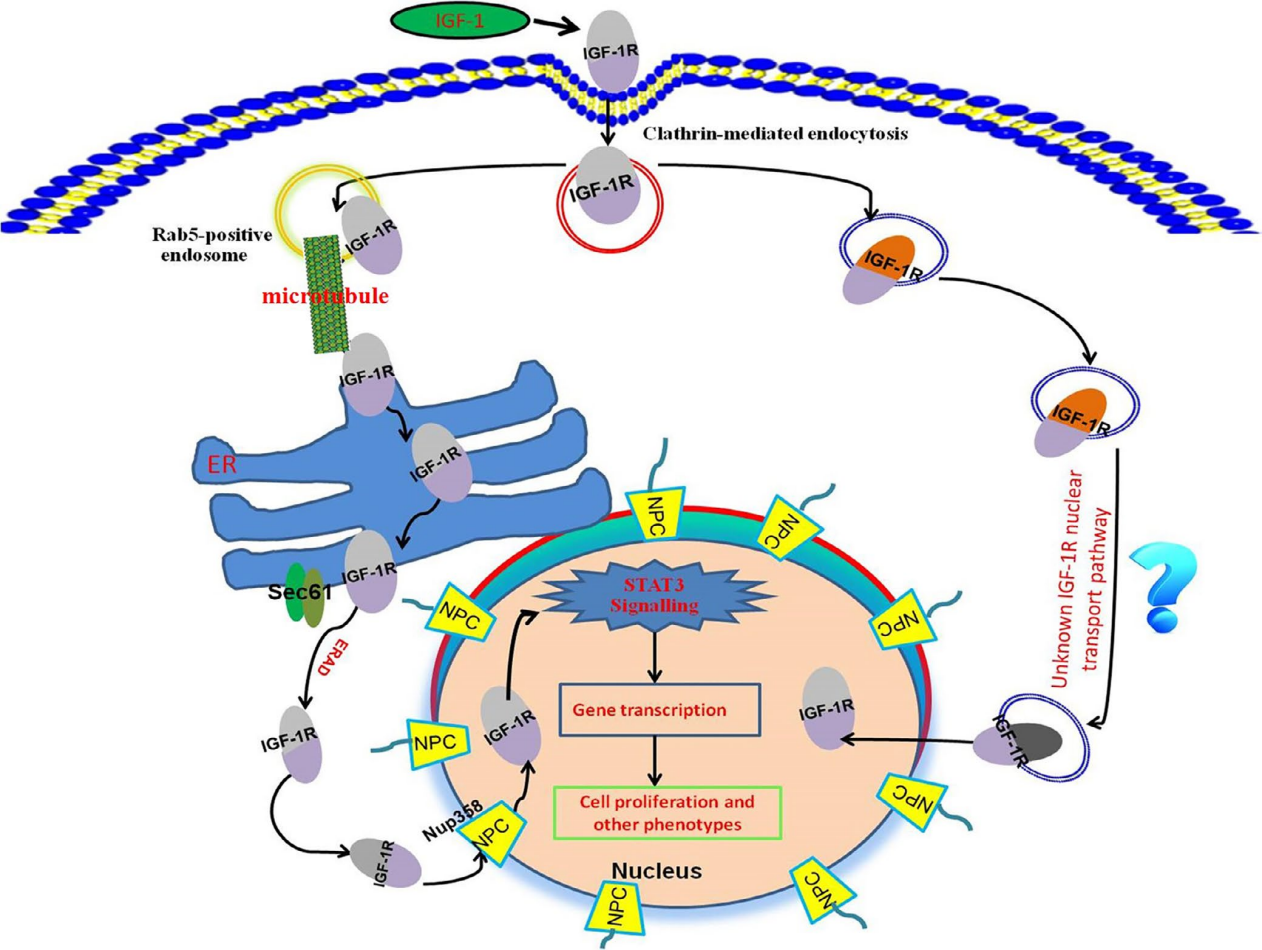
Molecular pathways of IGF‐1R’s nuclear localization revealed by current study

In the current study, we first discovered the IGF‐1R’s nuclear localization, revealed the basic pathway of IGF‐1R’s nuclear transport and found the potential functions and roles of nuclear‐localized IGF‐1R in the intestinal cell model, and we also proposed a possible nuclear transport pathway for IGF‐1R (Figure 11). Our current findings laid the foundation for related research in this field: 1) investigation of the roles of IGF‐1/IGF‐1R in the intestine cannot be only from the perspective of cell membranes, it should pay more attention to the functions of nuclear‐localized IGF‐1R; 2) currently, the biological functions of IGF‐1R localized in the intestinal nucleus are still a scientific ‘black‐box’, which requires further research to reveal the biological functions of nuclear‐localized IGF‐1R (although the current study suggest that IGF‐1R may act as a transcription enhancer though regulation of STAT3’s nuclear residence, the nuclear‐localized IGF‐1R must have more biological functions that need to be revealed, which implies that many phenotypes and functions related to IGF‐1R’s nuclear localization will be uncovered in the future based on our current work); 3) studies has shown that nuclear‐localized IGF‐1R is closely related with the occurrence and development of tumours; therefore, blocking the nuclear localization of IGF‐1R may become an anti‐tumour strategy. The current work revealed the basic pathway of IGF‐1R’s nuclear localization, which provides an important reference to identify targets that inhibit the nuclear localization of IGF‐1R; 4) current work showed that nuclear‐localized IGF‐1R is closely related to the hyperactivation of STAT3, and STAT3 itself is an important signalling molecule closely related with cell malignancy,^29^, ^30^, ^31^, ^32^ which suggests that nuclear‐localized IGF‐1R may be closely involved in the occurrence and development of intestinal tumours.

Taken together, the current research lays the foundation for further investigation of the functions and roles of nuclear‐localized IGF‐1/IGF‐1R on intestinal cells in the future. More importantly, we present the first study that proposes a basic pathway by which IGF‐1R transports into the cell nucleus, a new molecular mechanism by which nuclear‐localized IGF‐1R exhibited its potential bioactivities.

## 5 CONCLUSION

We revealed the molecular mechanism by which IGF‐1/IGF‐1R transports into the cell nuclei of intestinal cells. More importantly, the current work showed that the nuclear‐localized IGF‐1R has important biological functions by regulation of STAT3’ s subcellular localization and bioactivity.

## CONFLICT OF INTEREST

The authors declare no conflict of interest.

## AUTHOR CONTRIBUTIONS

XM, XH, YO and SY performed the experiments, and XM and XH wrote the manuscript. WJM conceived and supervised the whole project. All authors read and approved the final manuscript.

## Supporting information

Figure S1

Figure S2

Figure S3

Figure S4

Figure S5

Figure S6

Figure S7A

Figure S7B

Figure S8

Figure S9A

Figure S9B

Figure S9C

Figure S9D

Figure S9E

Figure S9F

Figure S9G

Figure S10

## Data Availability

All data sets generated for this study are included in the manuscript.
