# Supplementary figures and images for: The basic route of nuclear‐targeted transport of IGF‐1/IGF‐1R and potential biological functions in intestinal epithelial cells
Source: Cell Prolif. 2021 May 1;54(6):e13030. doi: 10.1111/cpr.13030 (PMC8168413; doi:10.1111/cpr.13030)

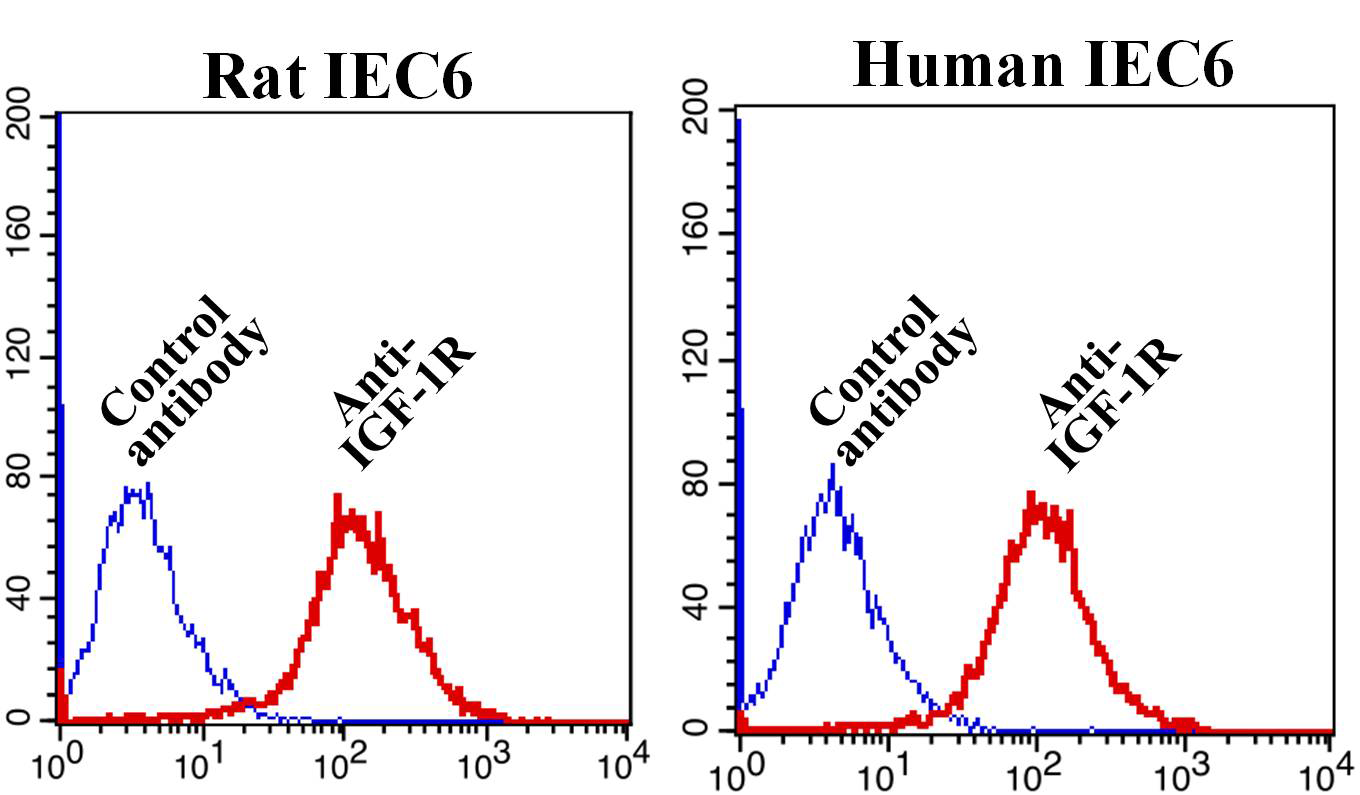

Supplement: Supplementary file 1 — Figure S1 [file CPR-54-e13030-s017.tif]

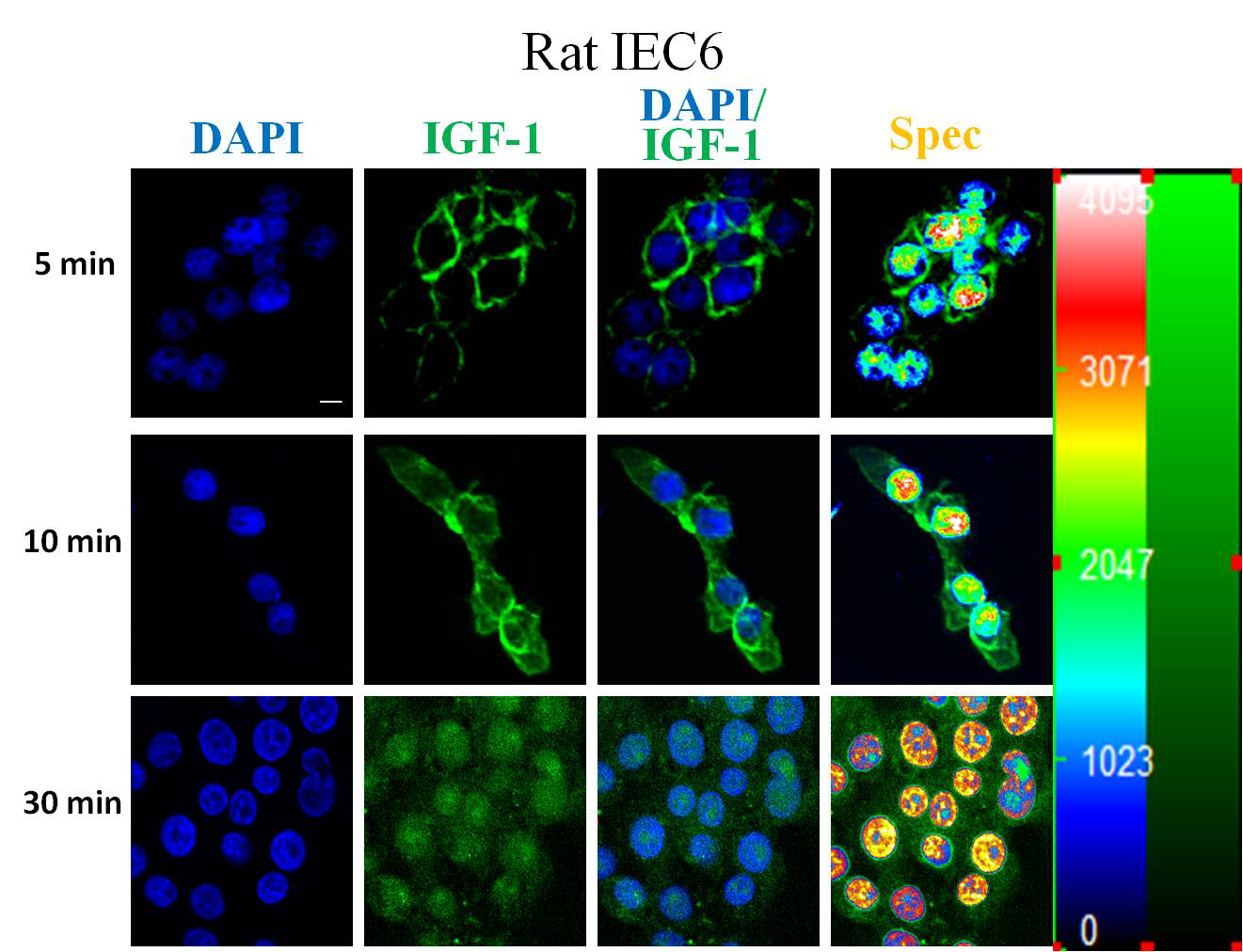

Supplement: Supplementary file 2 — Figure S2 [file CPR-54-e13030-s011.tif]

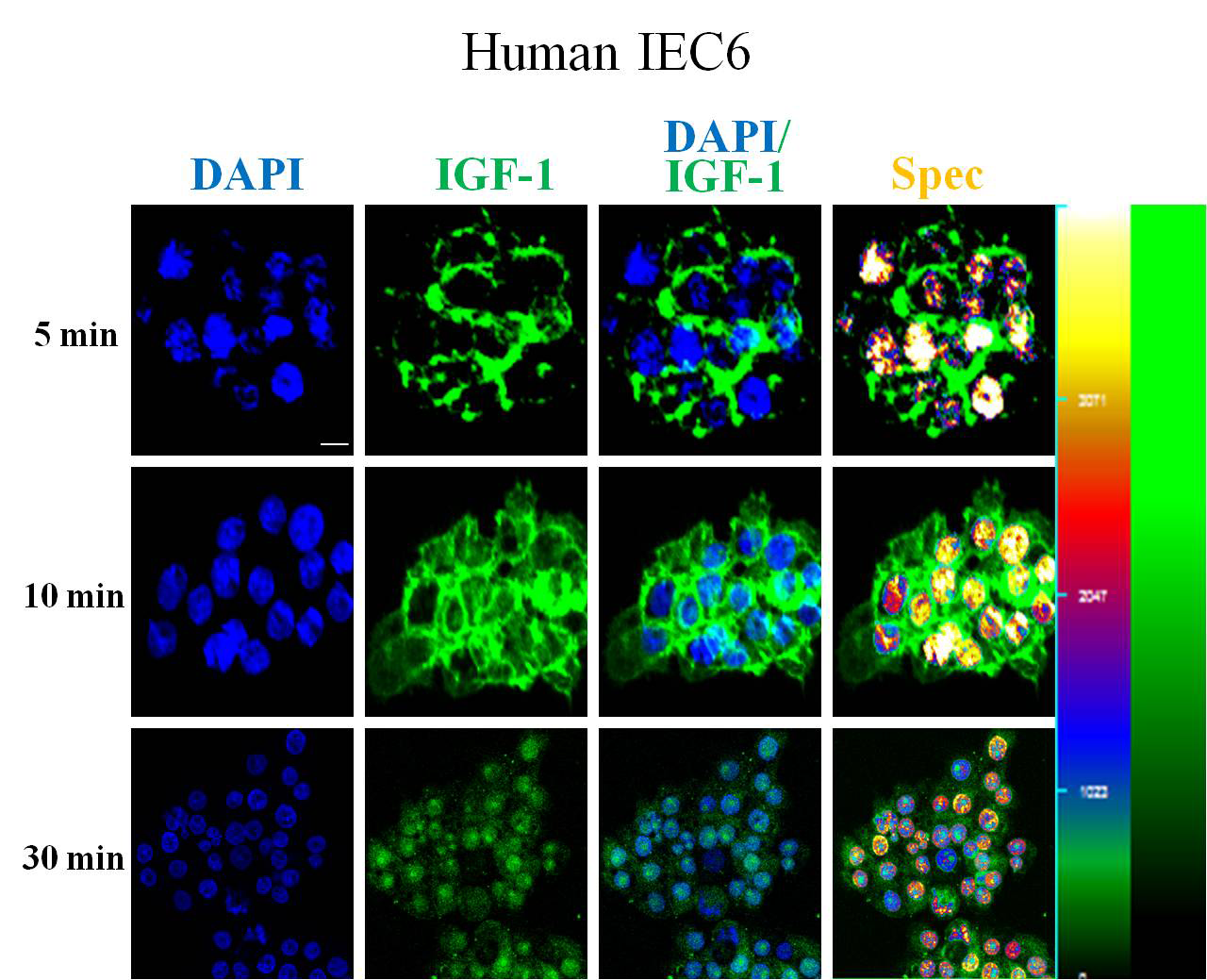

Supplement: Supplementary file 3 — Figure S3 [file CPR-54-e13030-s006.tif]

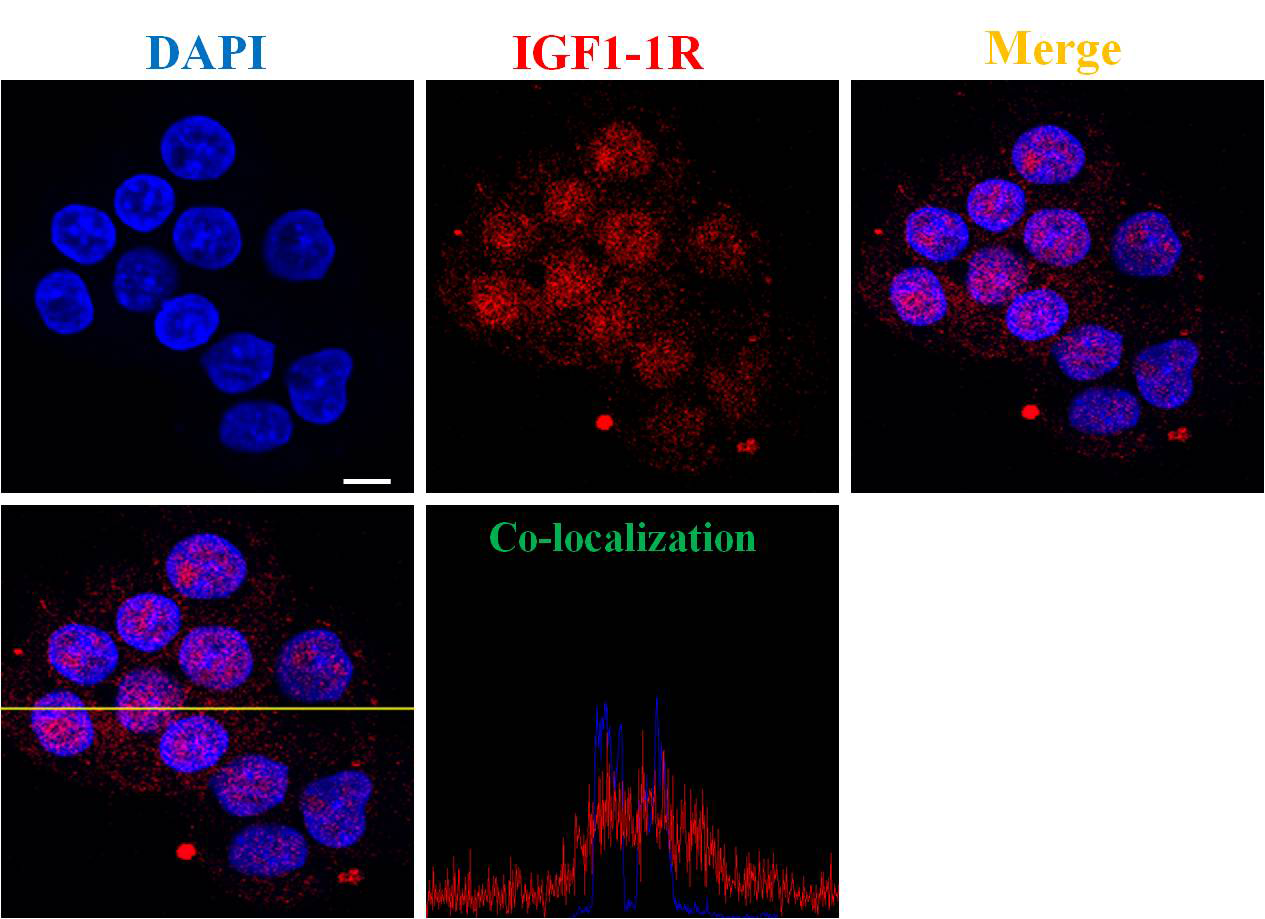

Supplement: Supplementary file 4 — Figure S4 [file CPR-54-e13030-s012.tif]

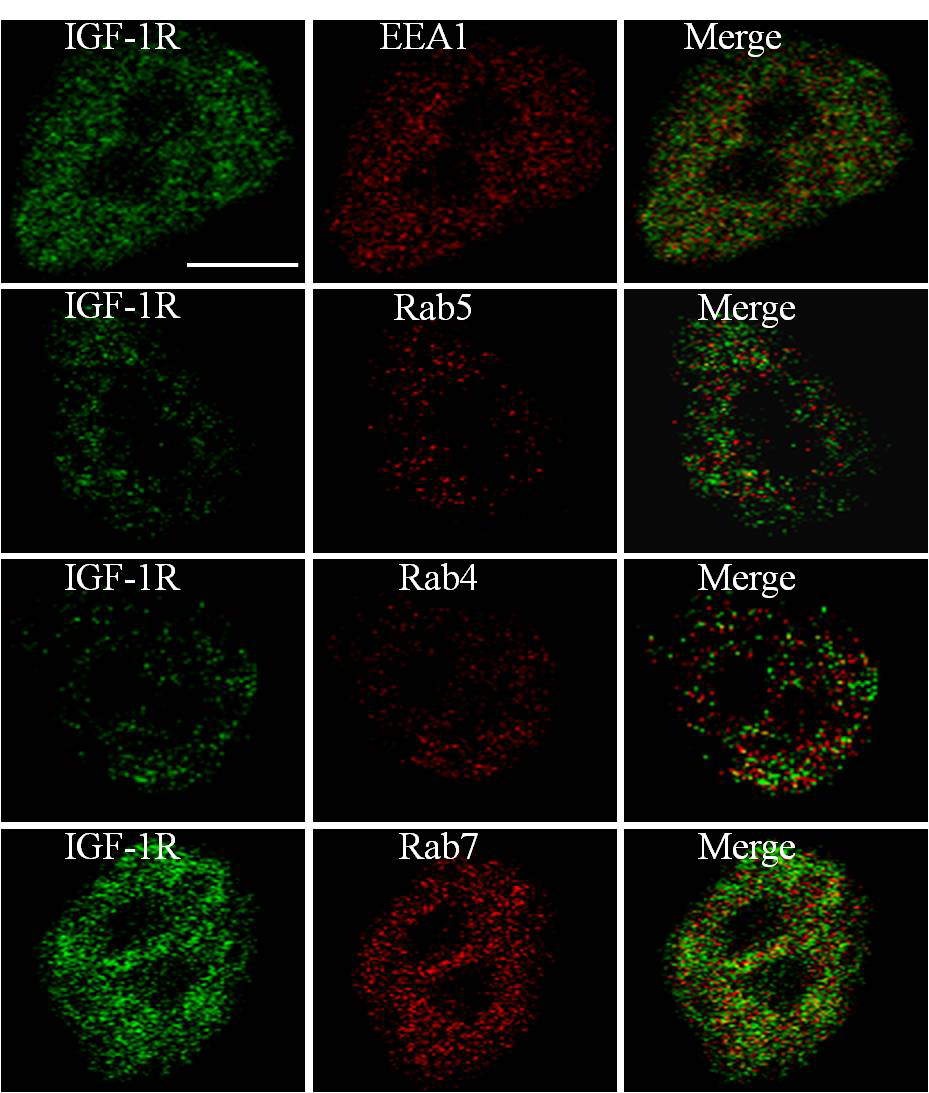

Supplement: Supplementary file 5 — Figure S5 [file CPR-54-e13030-s004.tif]

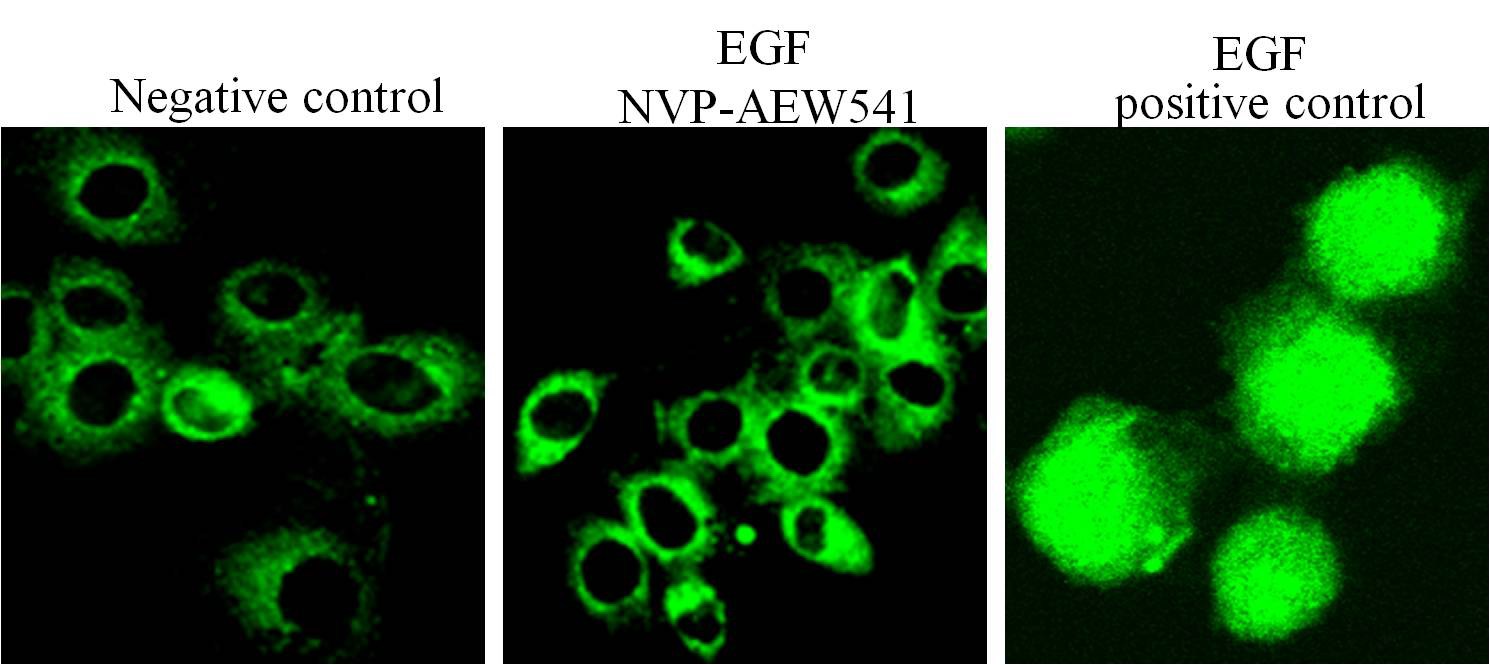

Supplement: Supplementary file 6 — Figure S6 [file CPR-54-e13030-s015.tif]

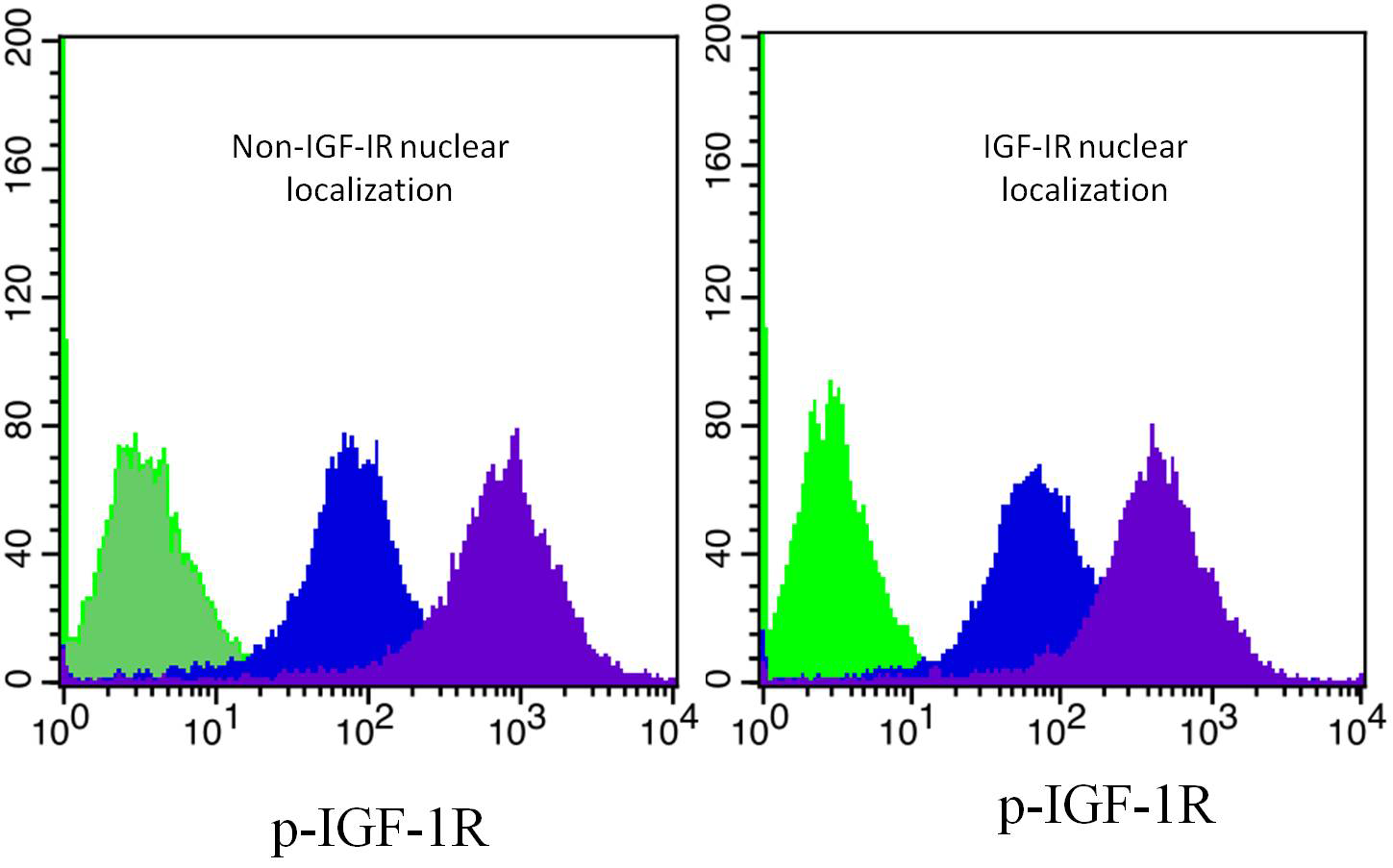

Supplement: Supplementary file 7 — Figure S7A [file CPR-54-e13030-s010.tif]

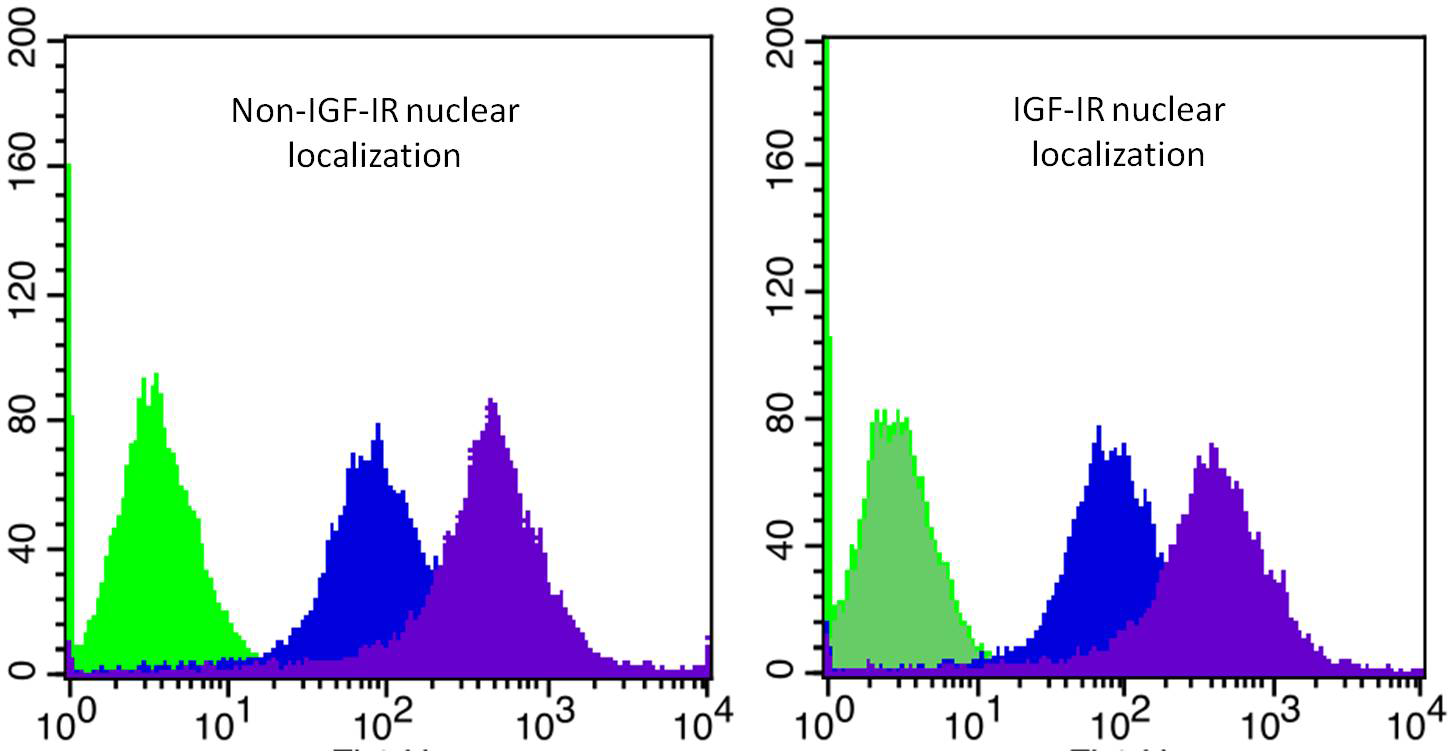

Supplement: Supplementary file 8 — Figure S7B [file CPR-54-e13030-s008.tif]

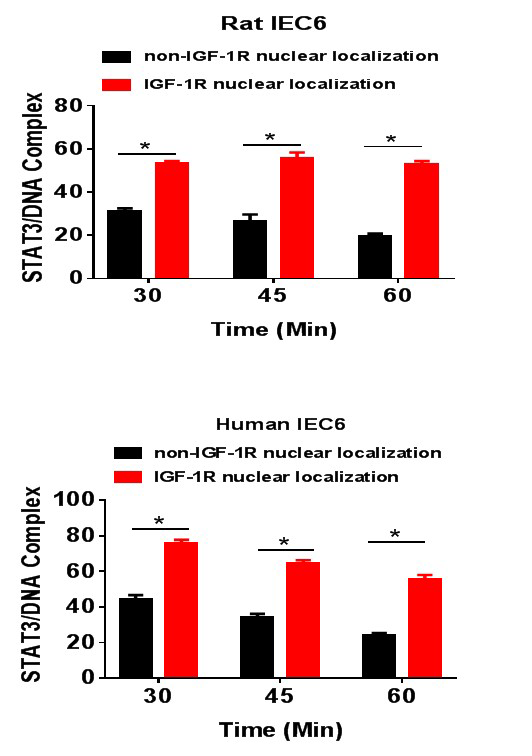

Supplement: Supplementary file 9 — Figure S8 [file CPR-54-e13030-s013.tif]

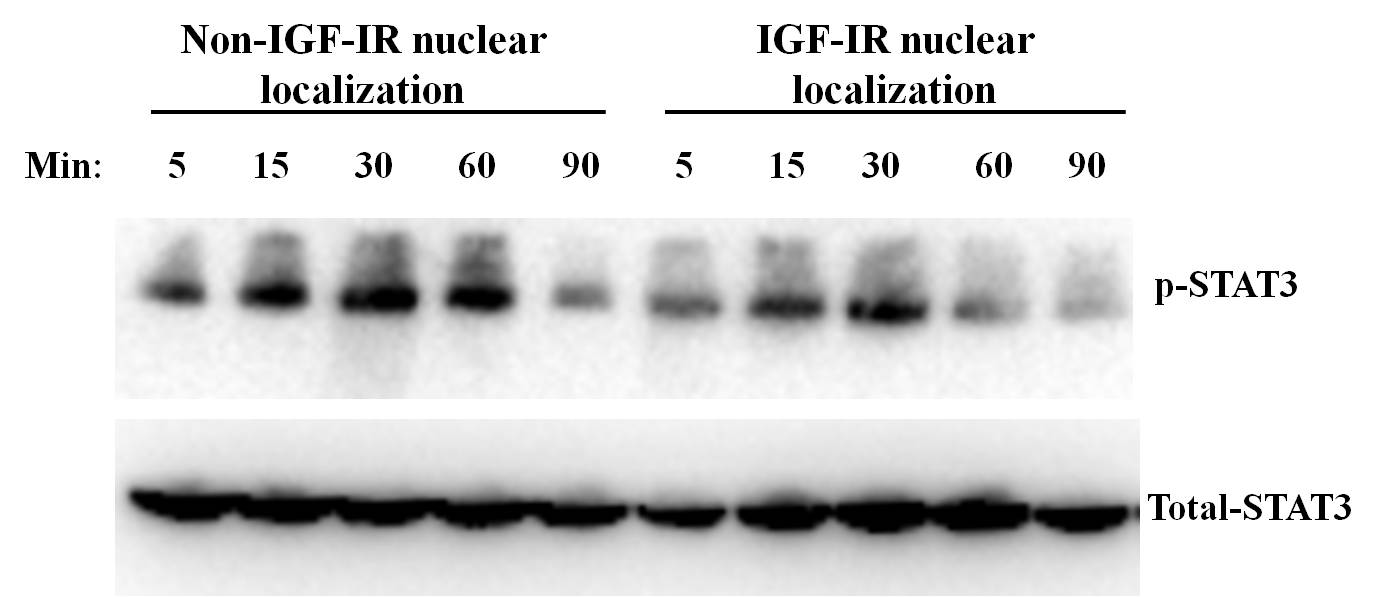

Supplement: Supplementary file 10 — Figure S9A [file CPR-54-e13030-s003.tif]

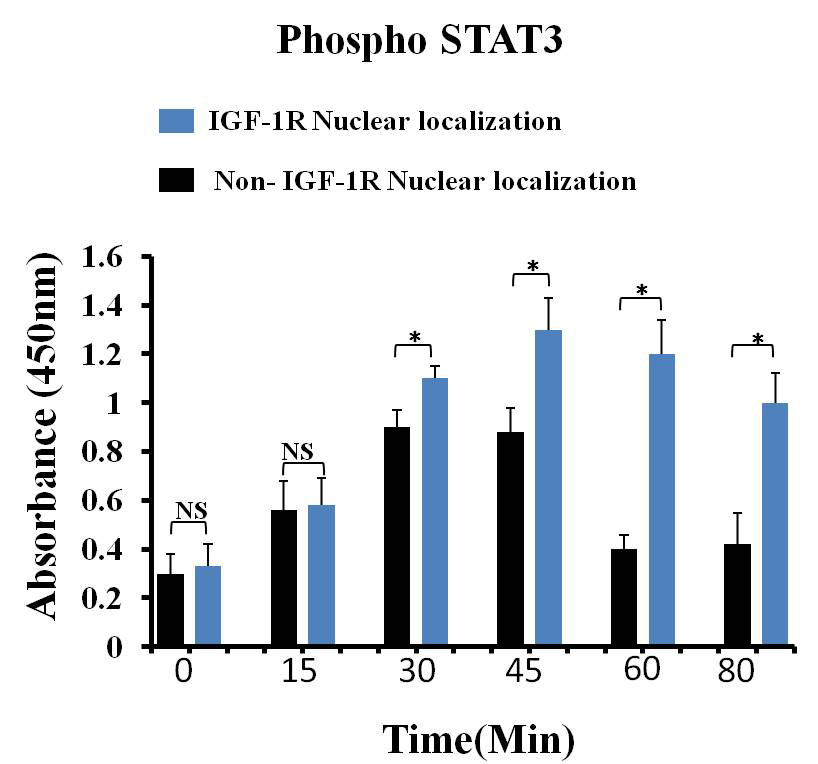

Supplement: Supplementary file 11 — Figure S9B [file CPR-54-e13030-s001.tif]

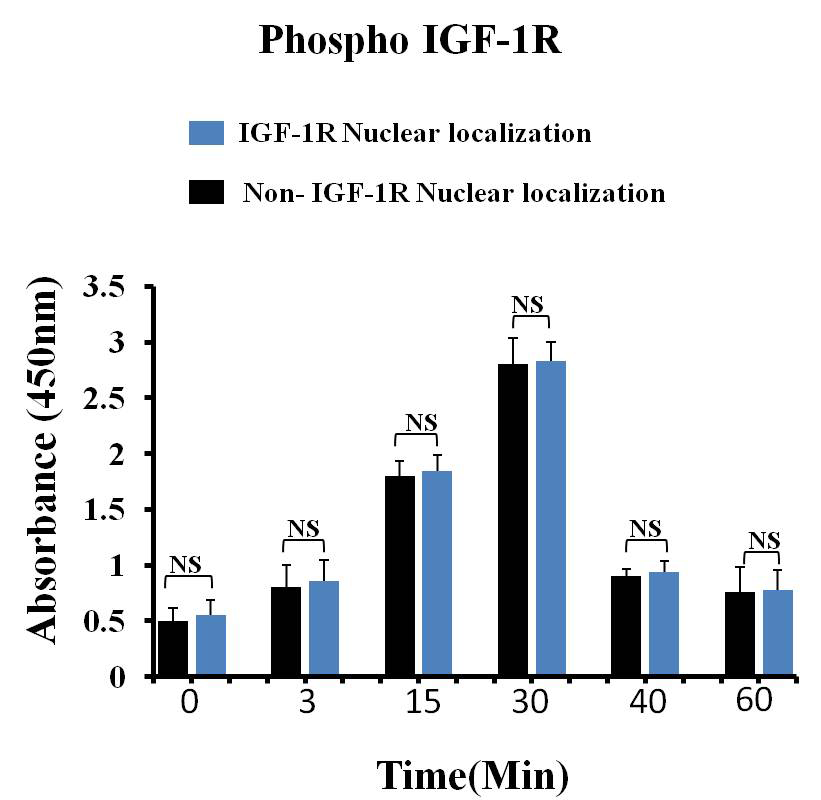

Supplement: Supplementary file 12 — Figure S9C [file CPR-54-e13030-s009.tif]

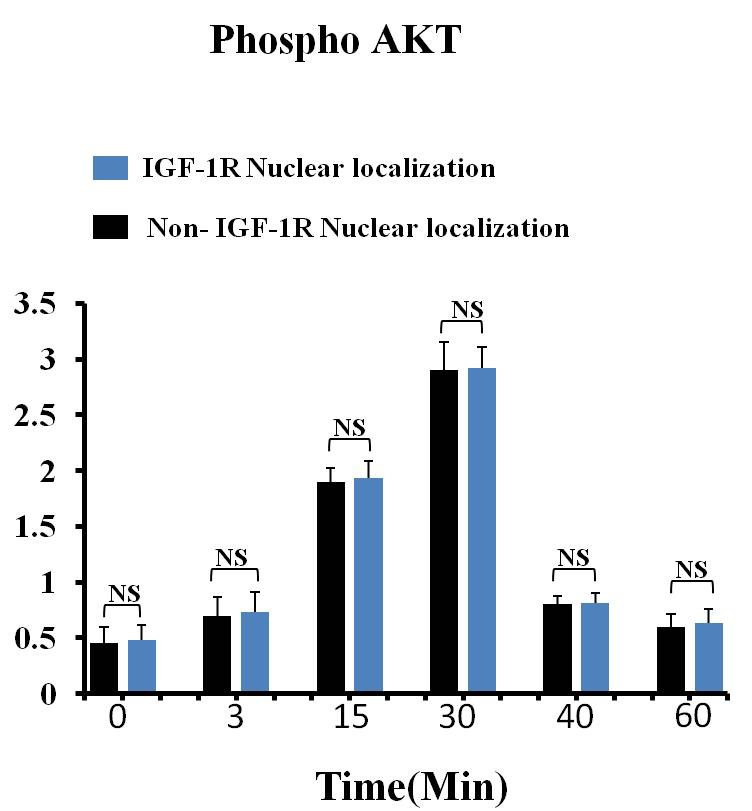

Supplement: Supplementary file 13 — Figure S9D [file CPR-54-e13030-s005.tif]

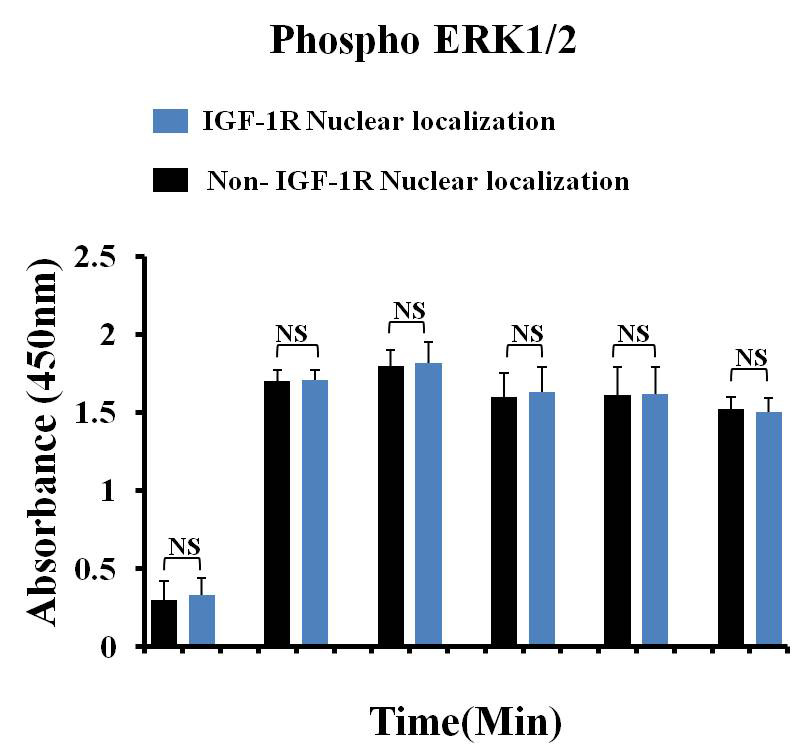

Supplement: Supplementary file 14 — Figure S9E [file CPR-54-e13030-s014.tif]

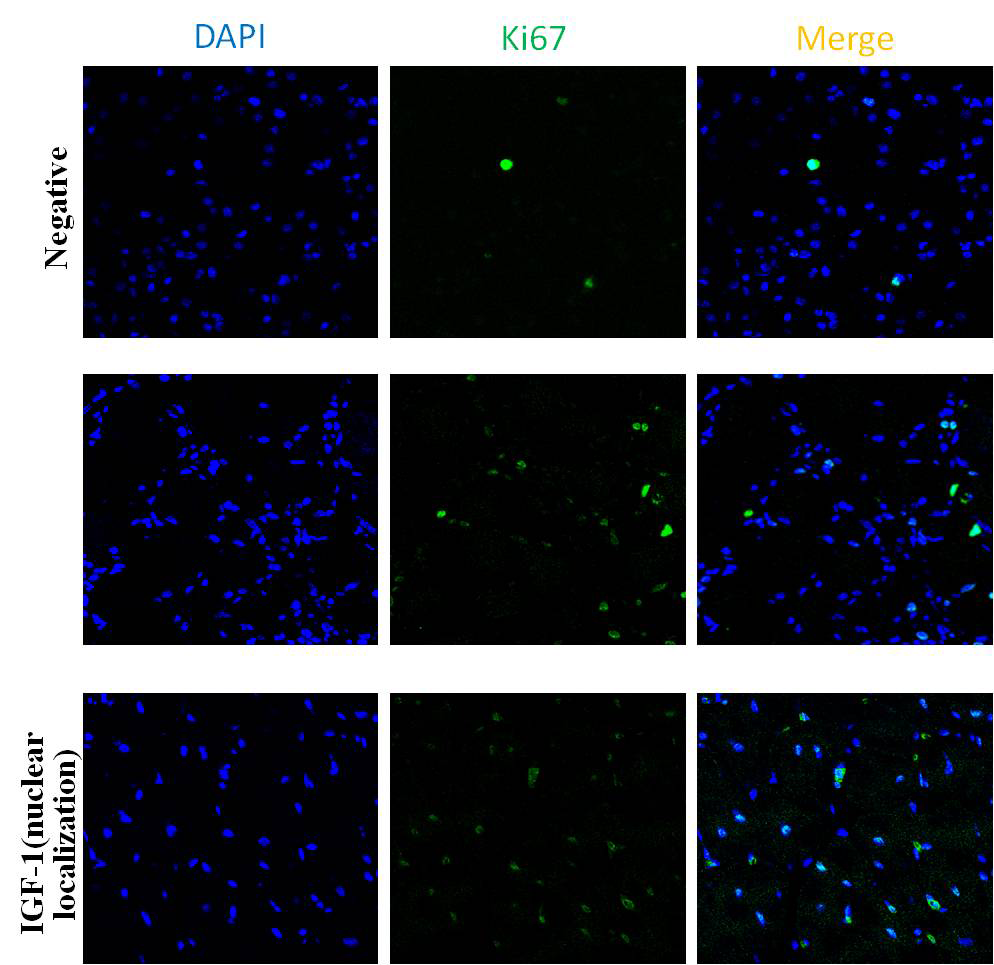

Supplement: Supplementary file 15 — Figure S9F [file CPR-54-e13030-s002.tif]

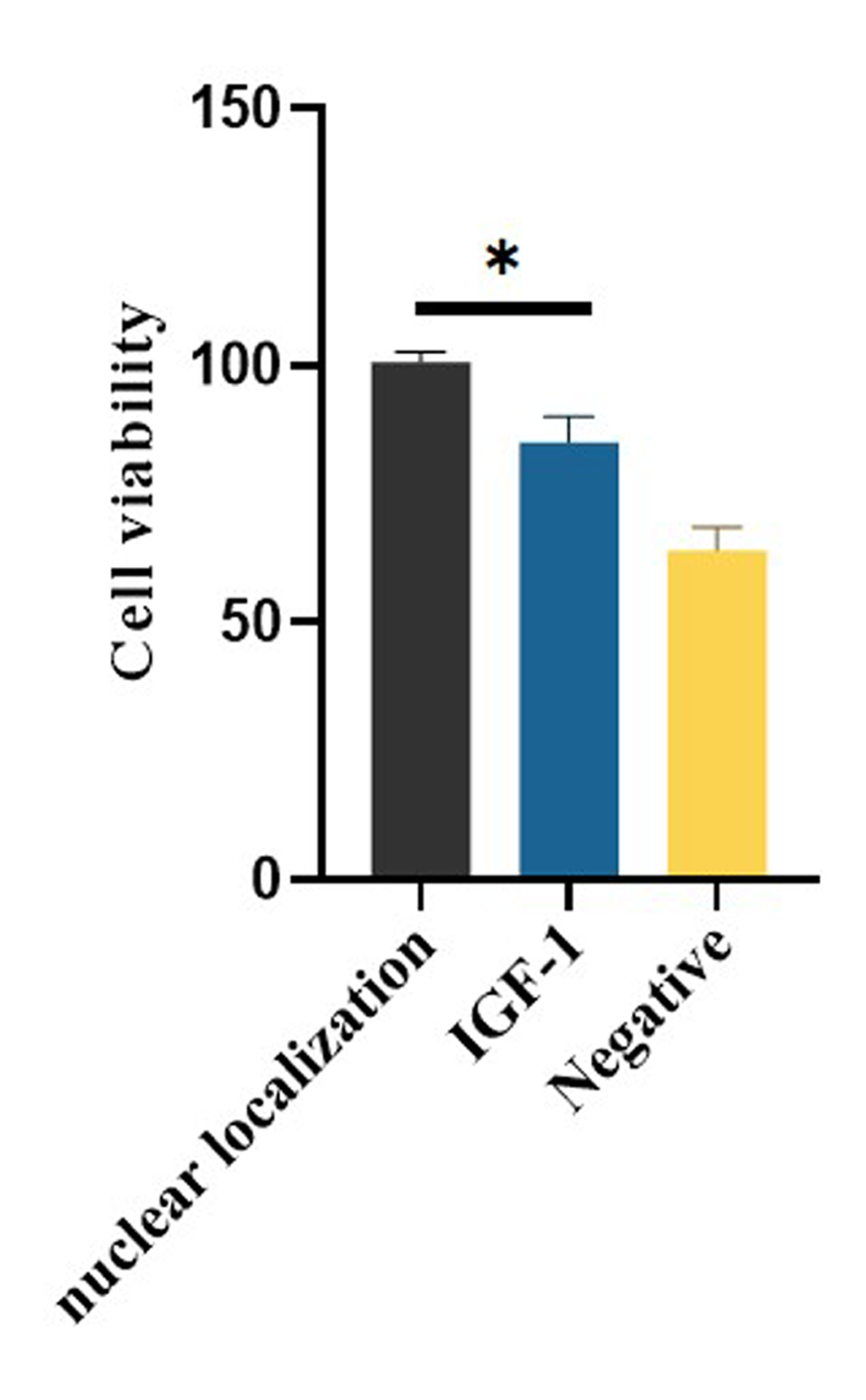

Supplement: Supplementary file 16 — Figure S9G [file CPR-54-e13030-s007.tif]

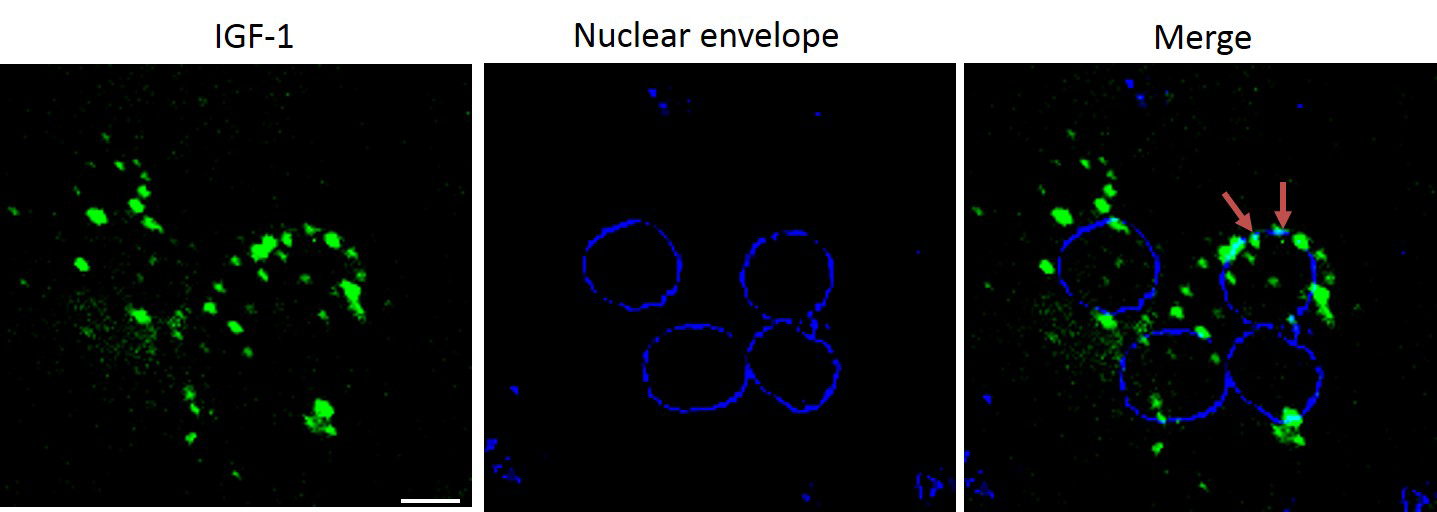

Supplement: Supplementary file 17 — Figure S10 [file CPR-54-e13030-s016.tif]
